# Supplementary material for: CNV-association meta-analysis in 191,161 European adults reveals new loci associated with anthropometric traits
Source: Nat Commun. 2017 Sep 29;8:744. doi: 10.1038/s41467-017-00556-x (PMC5622064; doi:10.1038/s41467-017-00556-x)
Supplement: Supplementary file 1 — Supplementary Information [file 41467_2017_556_MOESM1_ESM.pdf]

Description of Supplementary Files

File Name: Supplementary Information
Description: Supplementary Figures, Supplementary Tables, Supplementary Notes and Supplementary References

File Name: Supplementary Data 1
Description: All assessed probes with its frequency, effect size and P-value for BMI, weight, height and waist-to-hip ratio.

File Name: Peer Review File

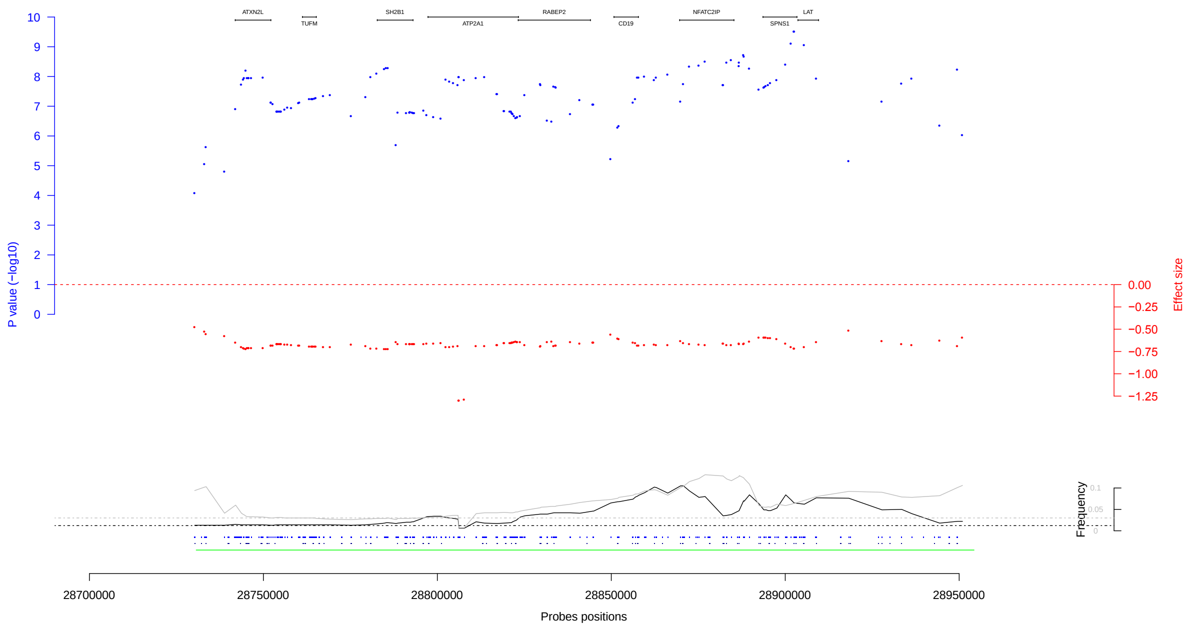

Supplementary Figure 1: Local Manhattan plot on the 16p11.2 220kb rearrangement. The blue dots correspond to the probes p-values of the association with the Weight. The red dots are the corresponding effect sizes. At the bottom the black and grey lines are the deletions and duplications frequencies. Finally, the dots at the bottom are the probes positions for the GIANT cohorts above and the UK Biobank below. The green line represents the known syndromic CNV in the region. Positions of the genes are set at the top of the plot.

A

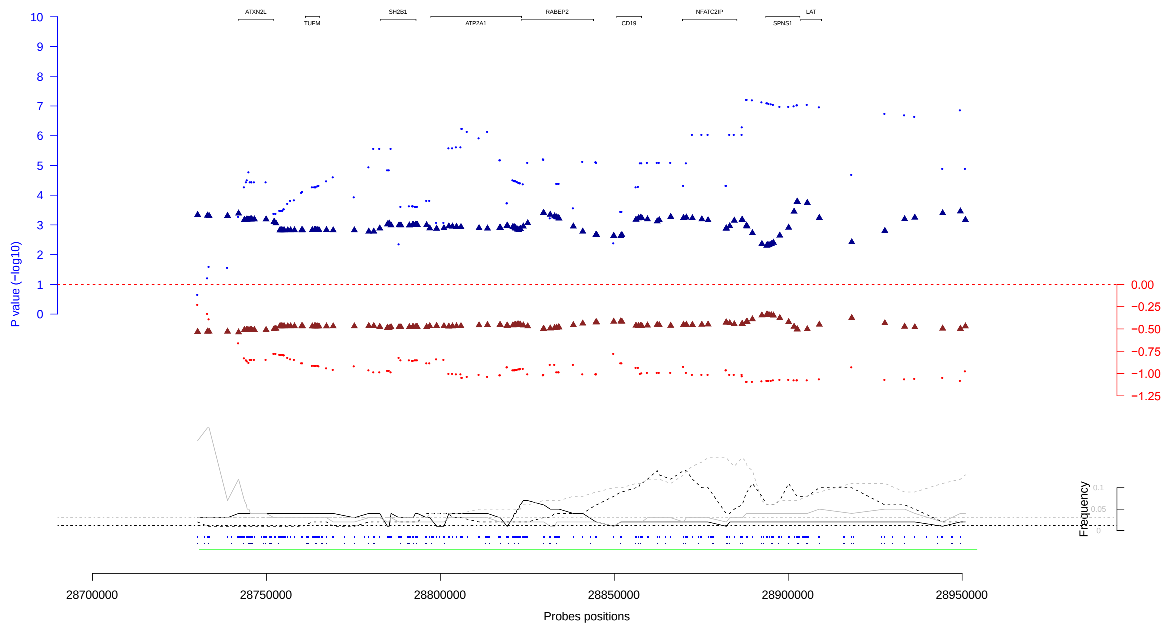

B

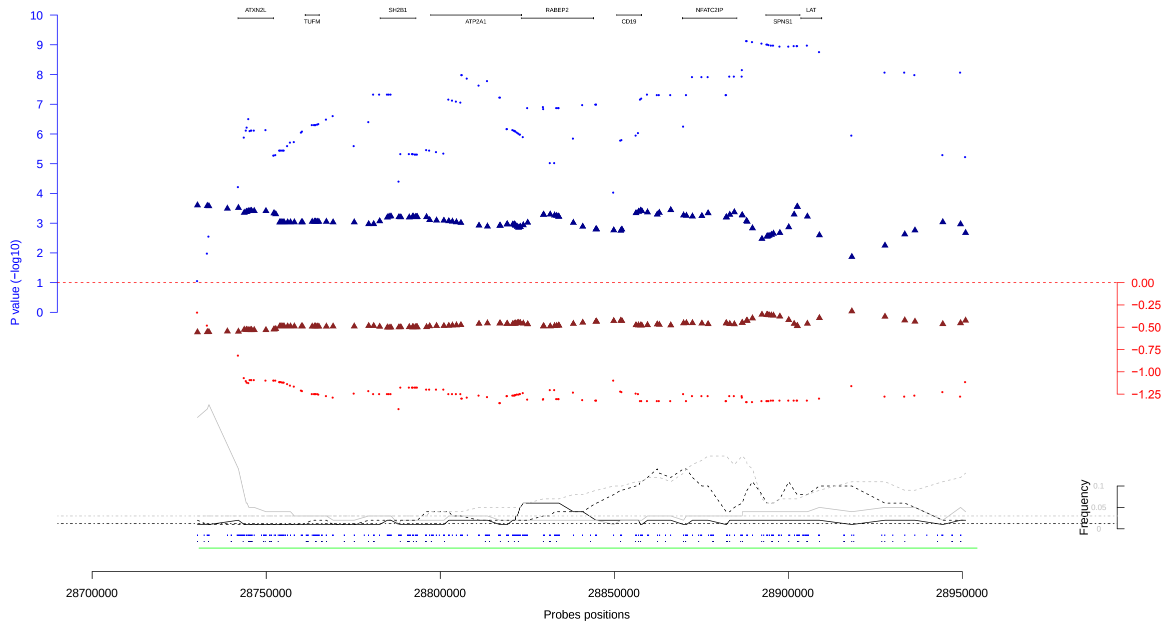

Supplementary Figure 2: (A) Local Manhattan plot on the 16p11.2 220kb rearrangement, separately for the GIANT data (dot) and the UKBB data (triangles). The blue dots/triangles correspond to the probes p-values of the association with the BMI. The red dots/triangles are the corresponding effect sizes. At the bottom the black and grey lines are the deletions and duplications frequencies. The plain one corresponds to the GIANT data while the dash

one to the UKBB. Finally, the dots at the bottom are the probes positions for the GIANT cohorts above and the UK Biobank below. The green line represents the known syndromic CNV in the region. Positions of the genes are set at the top of the plot. (**B)** Local Manhattan plot on the 16p11.2 220kb rearrangement, separately for the GIANT data (dot) and the UKBB data (triangles). The blue dots/triangles correspond to the probes p-values of the association with the Weight. The red dots/triangles are the corresponding effect sizes. At the bottom the black and grey lines are the deletions and duplications frequencies. The plain one corresponds to the GIANT data while the dash one to the UKBB. Finally, the dots at the bottom are the probes positions for the GIANT cohorts above and the UK Biobank below. The green line represents the known syndromic CNV in the region. Positions of the genes are set at the top of the plot.

A

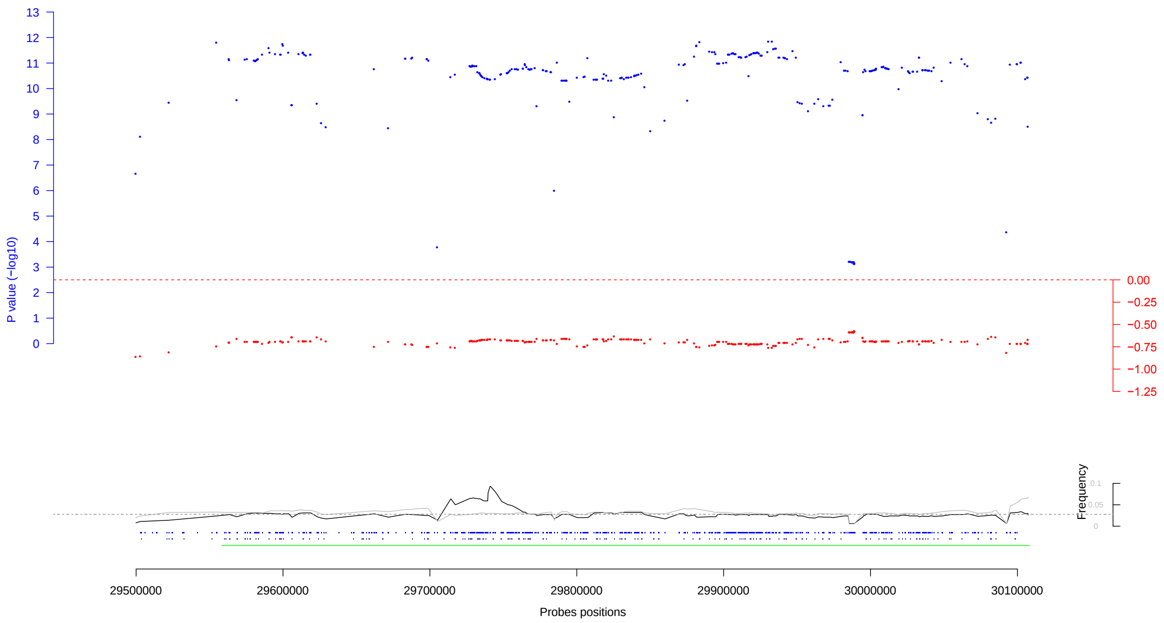
P value (−log10)
13
12
11
10
9
8
7
6
5
4
3
2
1
0
0.00
−0.25
−0.50
−0.75
−1.00
−1.25
Frequency
29500000
29600000
29700000
29800000
29900000
30000000
30100000
Probes positions

B

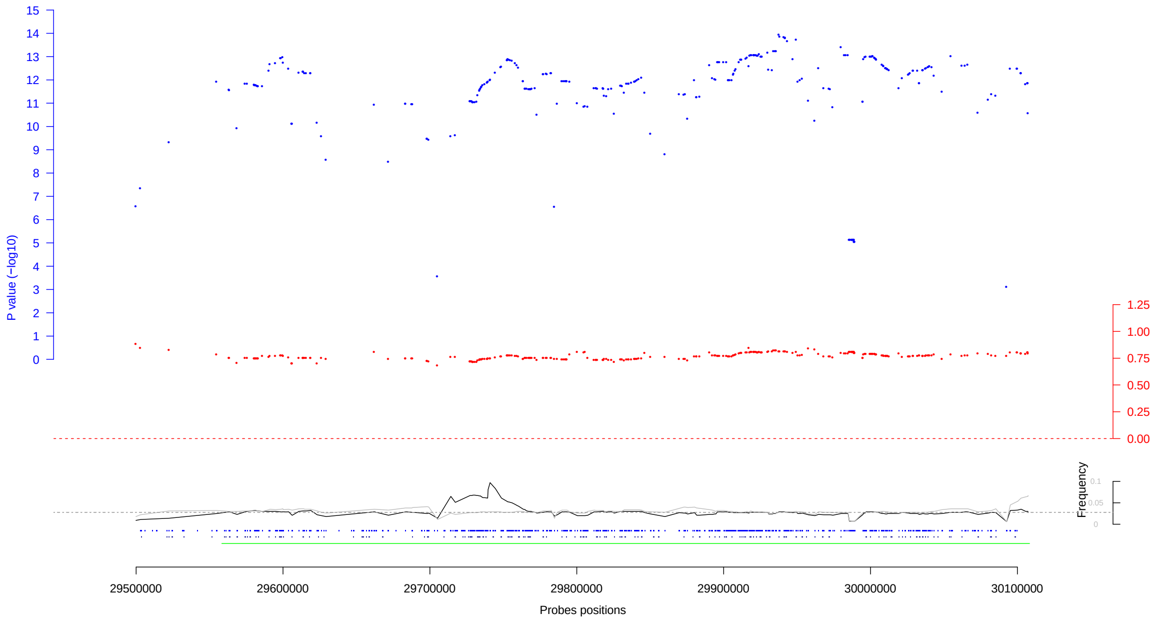
P value (−log10)
15
14
13
12
11
10
9
8
7
6
5
4
3
2
1
0
1.25
1.00
0.75
0.50
0.25
0.00
Frequency
29500000
29600000
29700000
29800000
29900000
30000000
30100000
Probes positions

C

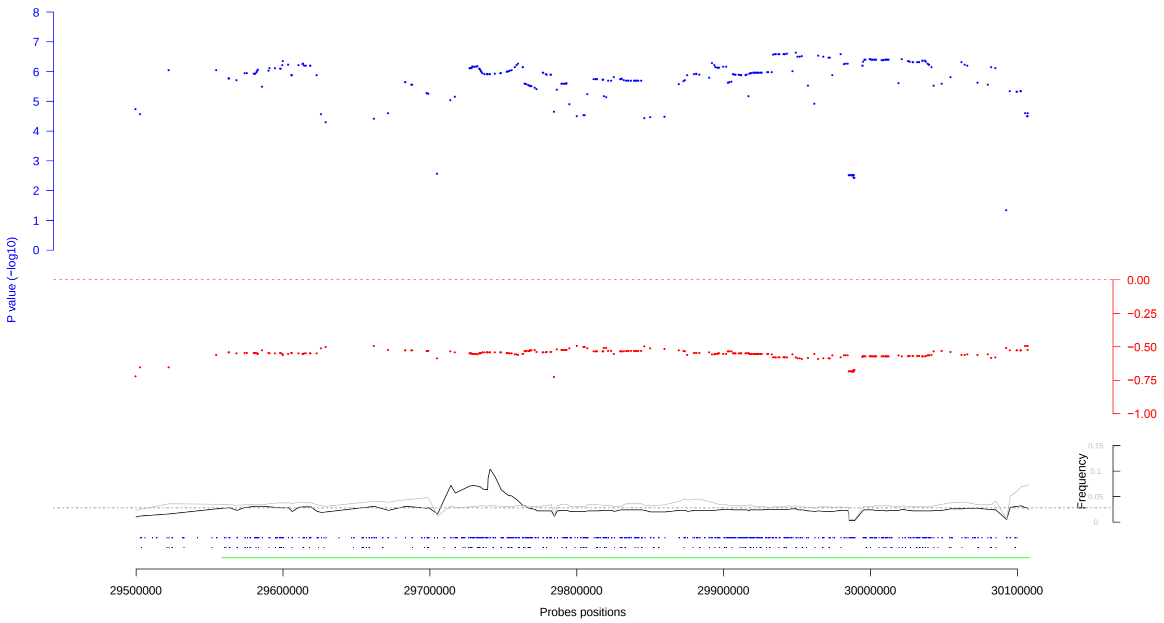

Supplementary Figure 3: (A) Local Manhattan plot on the 16p11.2 600kb rearrangement. The blue dots correspond to the probes p-values of the association with the BMI. The red dots are the corresponding effect sizes. At the bottom the black and grey lines are the deletions and duplications frequencies. Finally, the dots at the bottom are the probes positions for the GIANT cohorts above and the UK Biobank below. (**B)** Local Manhattan plot on the 16p11.2 600kb rearrangement. The blue dots correspond to the probes p-values of the association with the Height. The red dots are the corresponding effect sizes. At the bottom the black and grey lines are the deletions and duplications frequencies. Finally, the dots at the bottom are the probes positions for the GIANT cohorts above and the UK Biobank below. The green line represents the known syndromic CNV in the region. (**C)** Local Manhattan plot on the 16p11.2 600kb rearrangement. The blue dots correspond to the probes p-values of the association with the Waist-Hip ratio. The red dots are the corresponding effect sizes. At the bottom the black and grey lines are the deletions and duplications frequencies. Finally, the dots at the bottom are the probes positions for the GIANT cohorts above and the UK Biobank below. The green line represents the known syndromic CNV in the region.

A

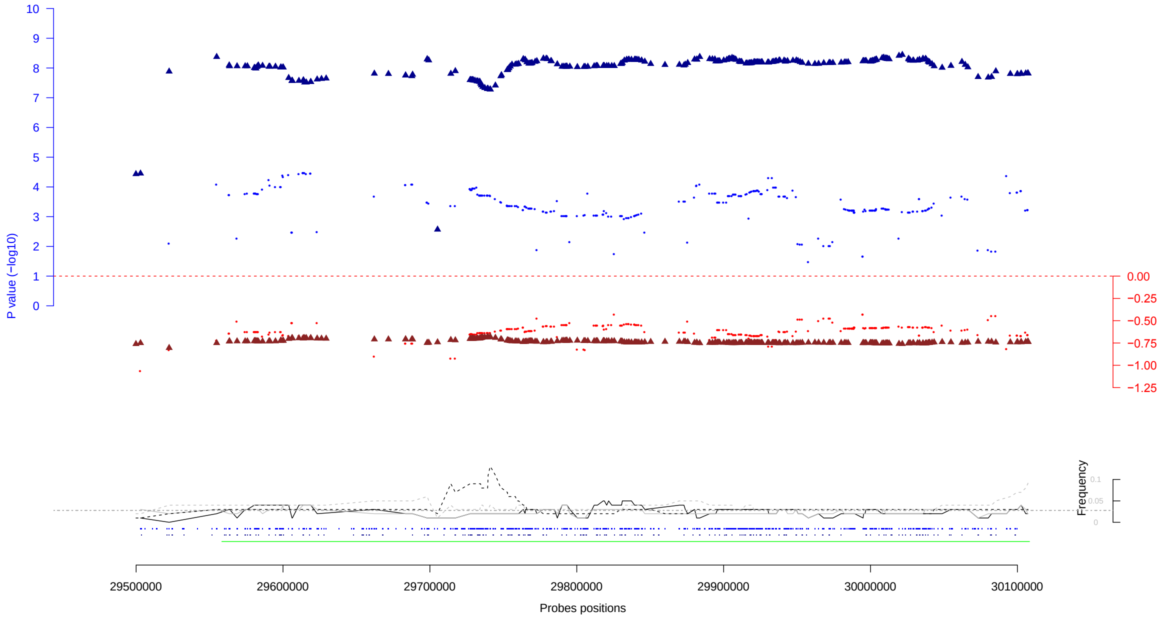
P value (−log10)
10
9
8
7
6
5
4
3
2
1
0
0.00
−0.25
−0.50
−0.75
−1.00
−1.25
Frequency
0.1
0.05
0
29500000
29600000
29700000
29800000
29900000
30000000
30100000
Probes positions

B

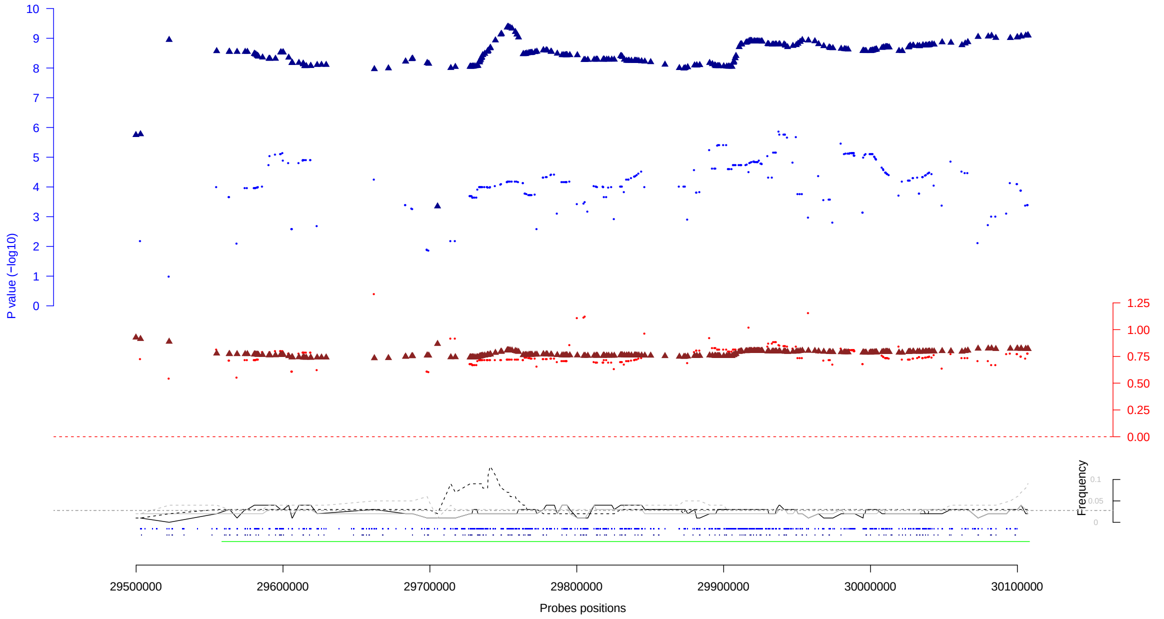
P value (−log10)
10
9
8
7
6
5
4
3
2
1
0
1.25
1.00
0.75
0.50
0.25
0.00
Frequency
0.1
0.05
0
29500000
29600000
29700000
29800000
29900000
30000000
30100000
Probes positions

C

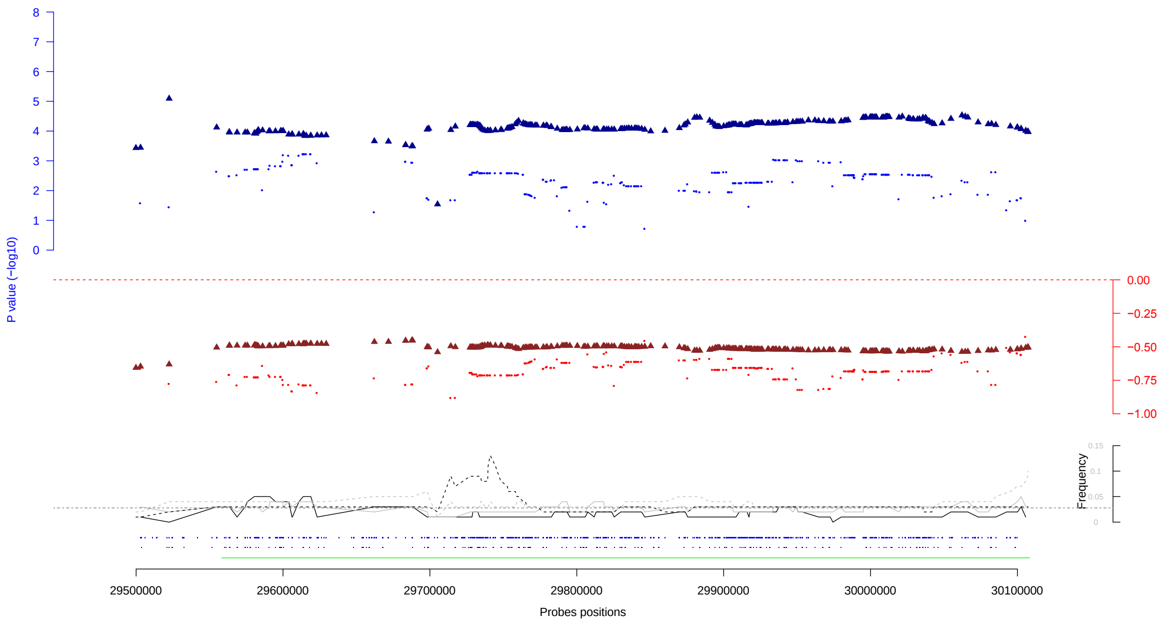

Supplementary Figure 4: (A) Local Manhattan plot on the 16p11.2 600kb rearrangement, separately for the GIANT data (dot) and the UKBB data (triangles). The blue dots/triangles correspond to the probes p-values of the association with the BMI. The red dots/triangles are the corresponding effect sizes. At the bottom the black and grey lines are the deletions and duplications frequencies. The plain one corresponds to the GIANT data while the dash one to the UKBB. Finally, the dots at the bottom are the probes positions for the GIANT cohorts above and the UK Biobank below. The green line represents the known syndromic CNV in the region. **(B)** Local Manhattan plot on the 16p11.2 600kb rearrangement, separately for the GIANT data (dot) and the UKBB data (triangles). The blue dots/triangles correspond to the probes p-values of the association with the Height. The red dots/triangles are the corresponding effect sizes. At the bottom the black and grey lines are the deletions and duplications frequencies. The plain one corresponds to the GIANT data while the dash one to the UKBB. Finally, the dots at the bottom are the probes positions for the GIANT cohorts above and the UK Biobank below. The green line represents the known syndromic CNV in the region. **(C)** Local Manhattan plot on the 16p11.2 600kb rearrangement, separately for the GIANT data (dot) and the UKBB data (triangles). The blue dots/triangles correspond to the probes p-values of the association with the Waist-Hip ratio. The red dots/triangles are the corresponding effect sizes. At the bottom the black and grey lines are the deletions and duplications frequencies. The plain one corresponds to the GIANT data while the dash one to the UKBB. Finally, the dots at the bottom are the probes positions for the GIANT cohorts above and the UK Biobank below. The green line represents the known syndromic CNV in the region.

A

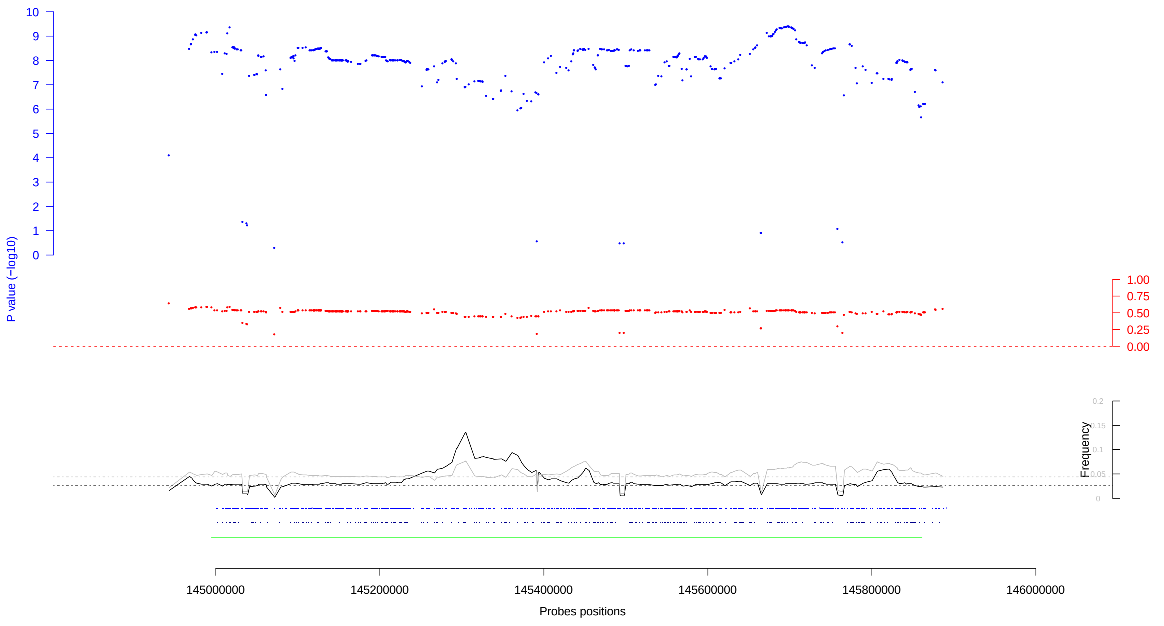

B

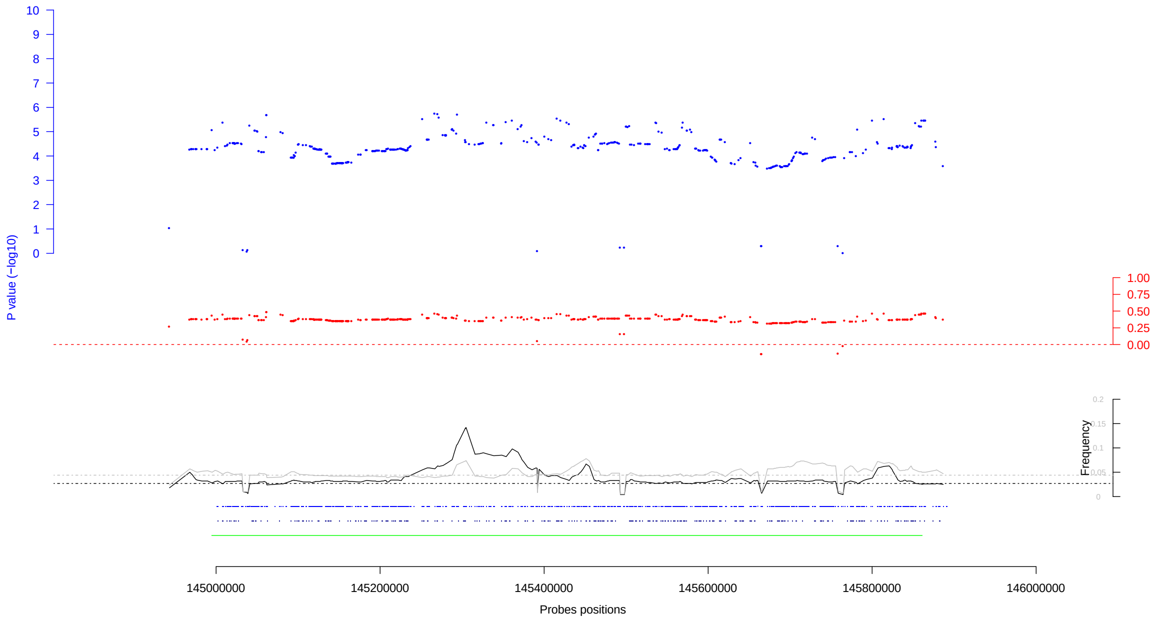

Supplementary Figure 5: (A) Local Manhattan plot on the 1p36 rearrangement. The blue dots correspond to the probes p-values of the association with the Height. The red dots are the corresponding effect sizes. At the bottom the black and grey lines are the deletions and duplications frequencies. Finally, the dots at the bottom are the probes positions for the GIANT cohorts above and the UK Biobank below. The green line represents the known

syndromic CNV in the region. Positions of the genes are set at the top of the plot. (**B)** Local Manhattan plot on the 1p36 rearrangement. The blue dots correspond to the probes p-values of the association with the Weight. The red dots are the corresponding effect sizes. At the bottom the black and grey lines are the deletions and duplications frequencies. Finally, the dots at the bottom are the probes positions for the GIANT cohorts above and the UK Biobank below. Positions of the genes are set at the top of the plot.

A

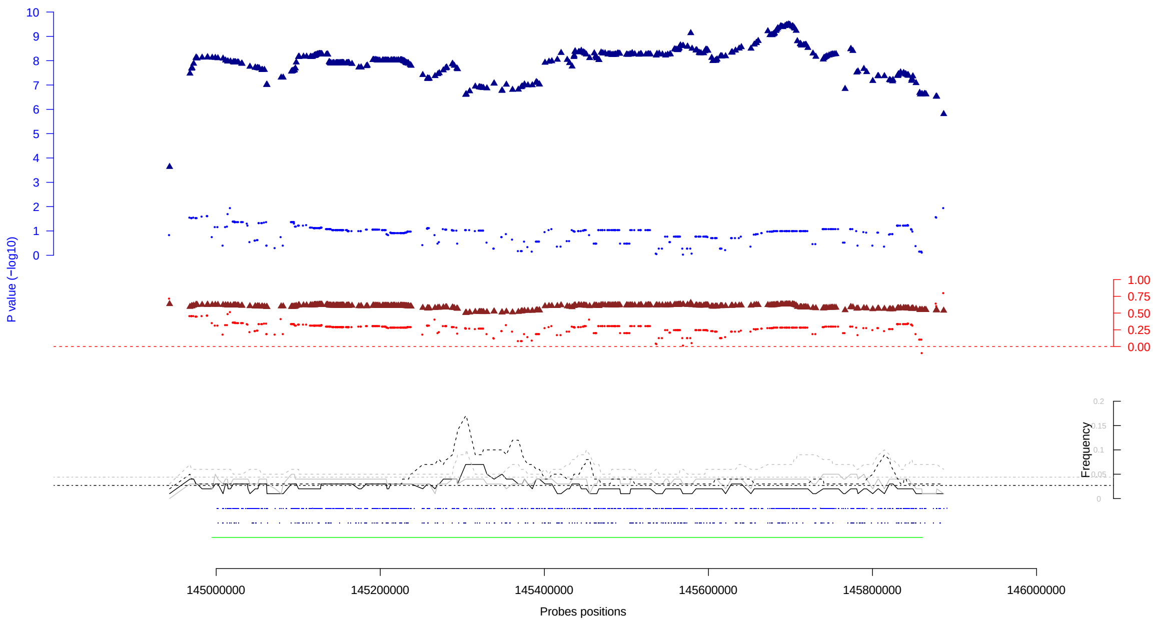

B

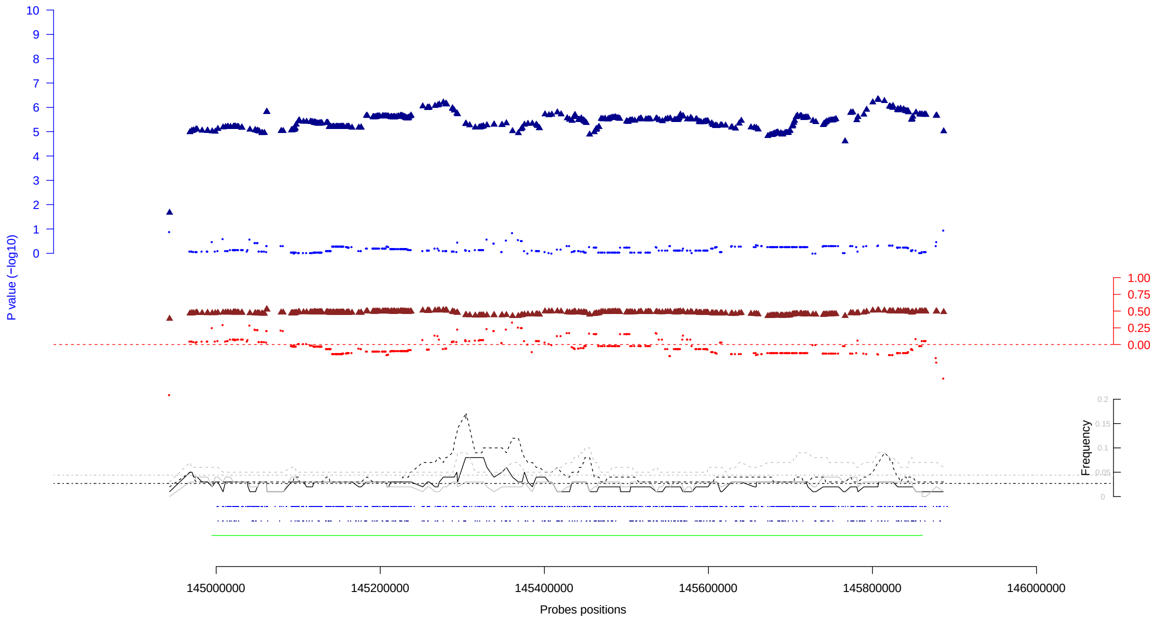

Supplementary Figure 6: (A) Local Manhattan plot on the 1p36 rearrangement, separately for the GIANT data (dot) and the UKBB data (triangles). The blue dots/triangles correspond to the probes p-values of the association with the Height. The red dots/triangles are the corresponding effect sizes. At the bottom the black and grey lines are the deletions and duplications frequencies. The plain one corresponds to the GIANT data while the dash one

to the UKBB. Finally, the dots at the bottom are the probes positions for the GIANT cohorts above and the UK Biobank below. The green line represents the known syndromic CNV in the region. Positions of the genes are set at the top of the plot. (**B)** Local Manhattan plot on the 1p36 rearrangement, separately for the GIANT data (dot) and the UKBB data (triangles). The blue dots/triangles correspond to the probes p-values of the association with the Weight. The red dots/triangles are the corresponding effect sizes. At the bottom the black and grey lines are the deletions and duplications frequencies. The plain one corresponds to the GIANT data while the dash one to the UKBB. Finally, the dots at the bottom are the probes positions for the GIANT cohorts above and the UK Biobank below. The green line represents the known syndromic CNV in the region. Positions of the genes are set at the top of the plot.

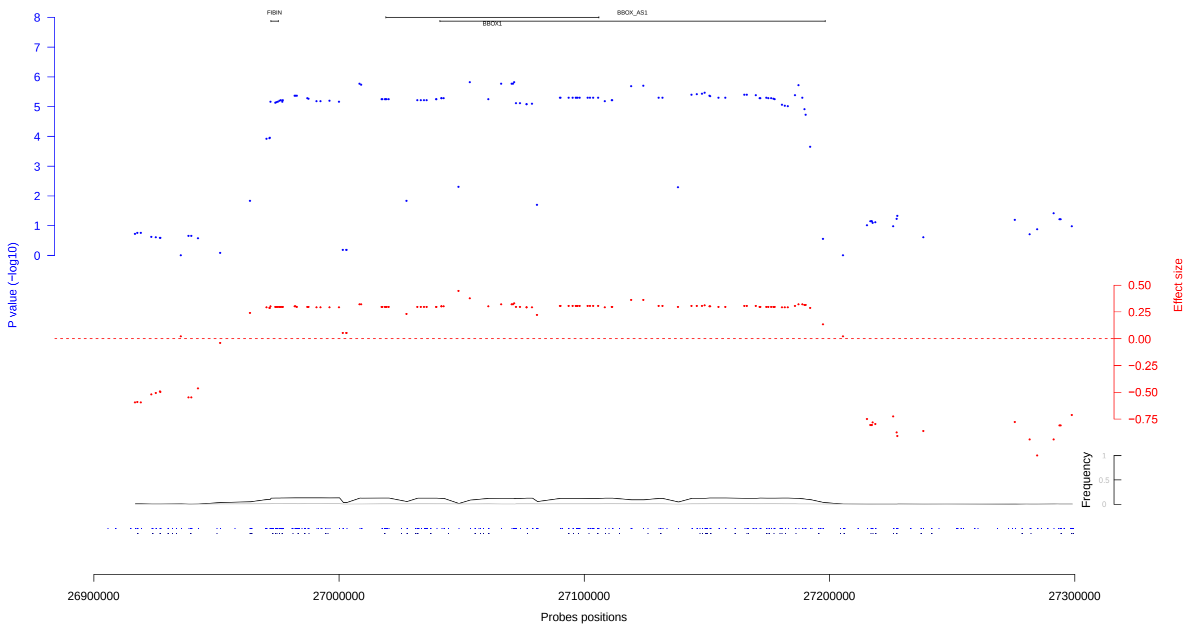

Supplementary Figure 7: Local Manhattan plot on the rearrangement near BBOX1 and Fibin. The blue dots correspond to the probes p-values of the association with the Height. The red dots are the corresponding effect sizes. At the bottom the black and grey lines are the deletions and duplications frequencies. Finally, the dots at the bottom are the probes positions for the GIANT cohorts above and the UK Biobank below. Positions of the genes are set at the top of the plot.

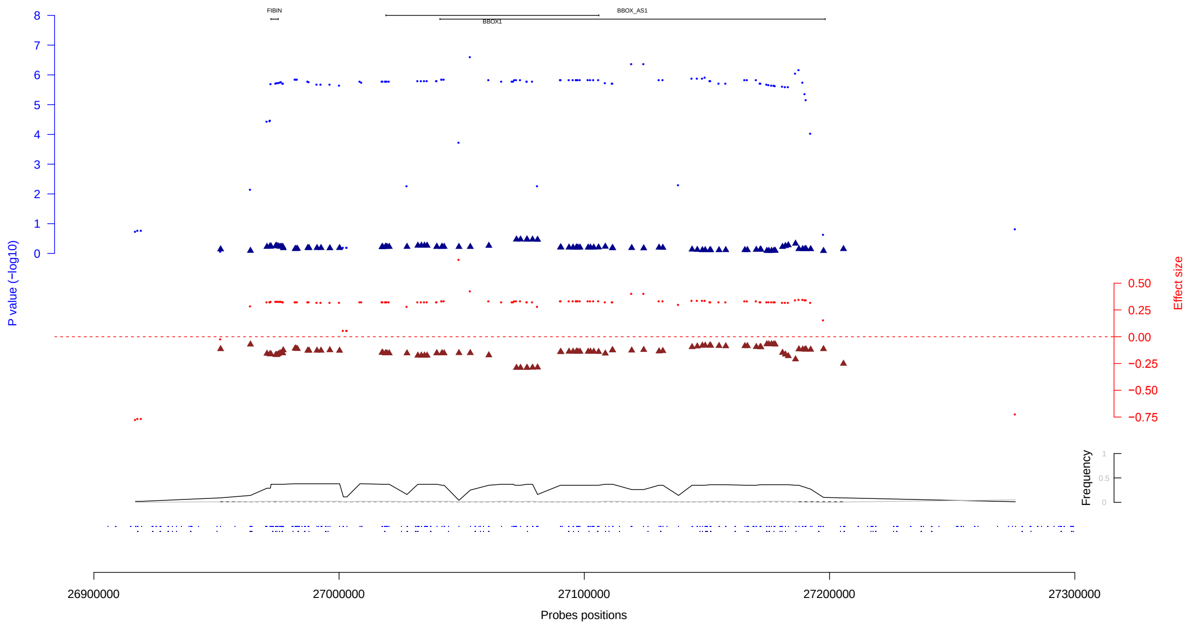

Supplementary Figure 8: Local Manhattan plot on the rearrangement near BBOX1 and Fibin, separately for the GIANT data (dot) and the UKBB data (triangles). The blue dots/triangles correspond to the probes p-values of the association with the Height. The red dots/triangles are the corresponding effect sizes. At the bottom the black and grey lines are the deletions and duplications frequencies. The plain one corresponds to the GIANT data while the dash one to the UKBB. Finally, the dots at the bottom are the probes positions for the GIANT cohorts above and the UK Biobank below. Positions of the genes are set at the top of the plot.

A

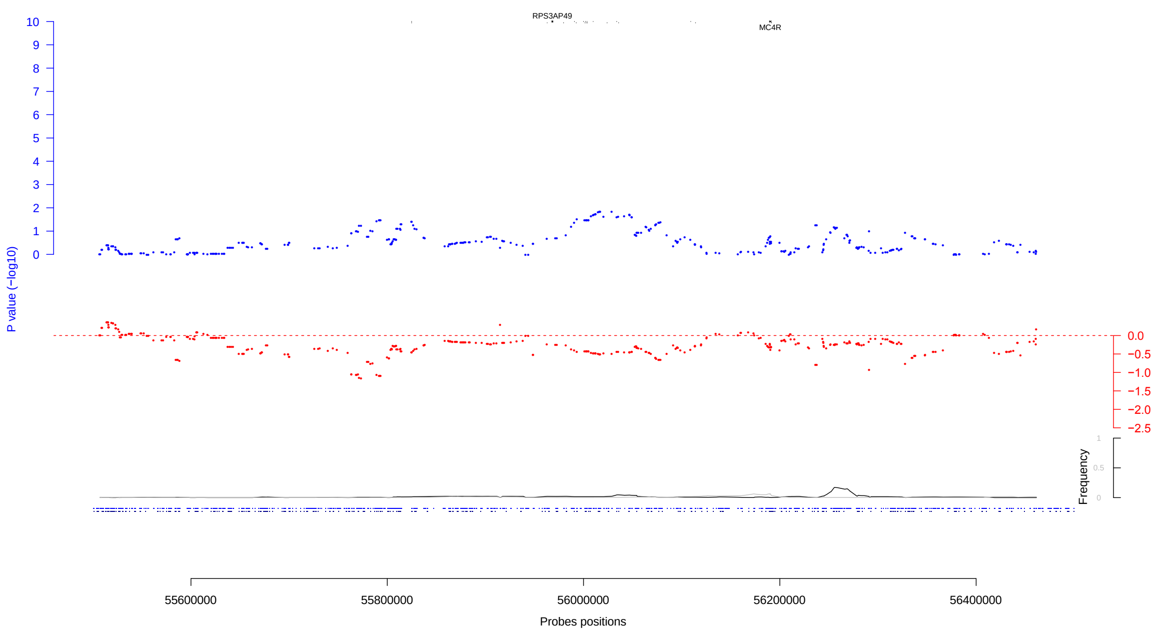

B

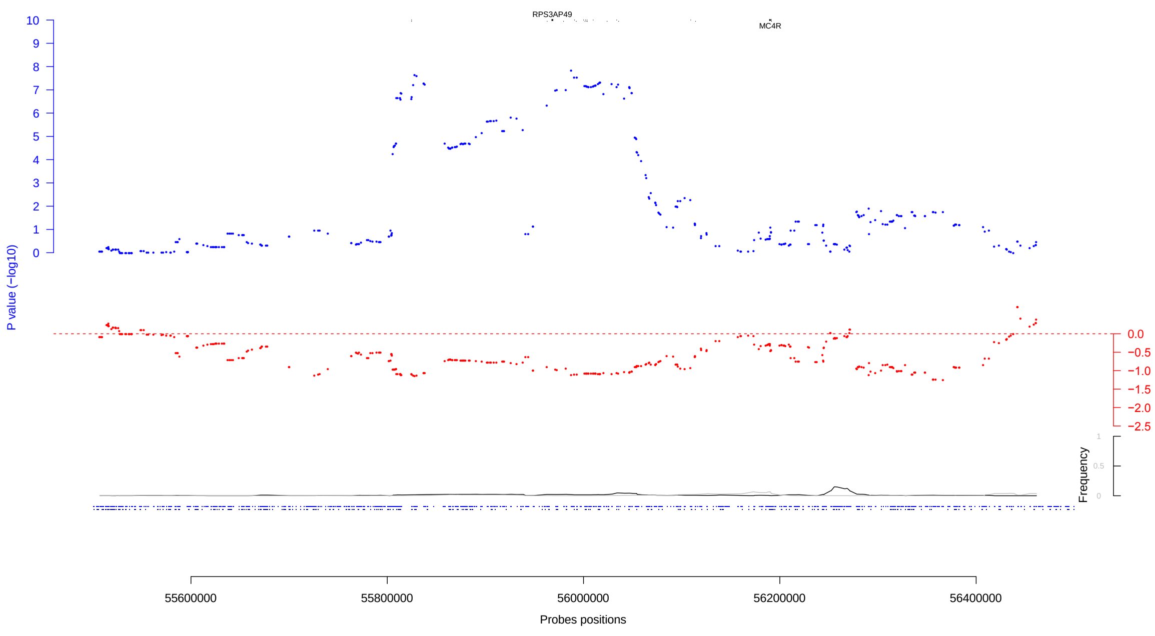

Supplementary Figure 9: (A) Local Manhattan plot on the rearrangement near MC4R. The blue dots correspond to the probes p-values of the association with the Height. The red dots are the corresponding effect sizes. At the bottom the black and grey lines are the deletions and duplications frequencies. Finally, the dots at the bottom are the probes positions for the GIANT cohorts above and the UK Biobank below. The position of the MC4R gene is set at

the top of the plot with the position of the BMI associated probes from GWAS. (**B)** Local Manhattan plot on the rearrangement near MC4R. The blue dots correspond to the probes p-values of the association with the Weight. The red dots are the corresponding effect sizes. At the bottom the black and grey lines are the deletions and duplications frequencies. Finally, the dots at the bottom are the probes positions for the GIANT cohorts above and the UK Biobank below. The position of the MC4R gene is set at the top of the plot with the position of the BMI associated probes from GWAS.

A

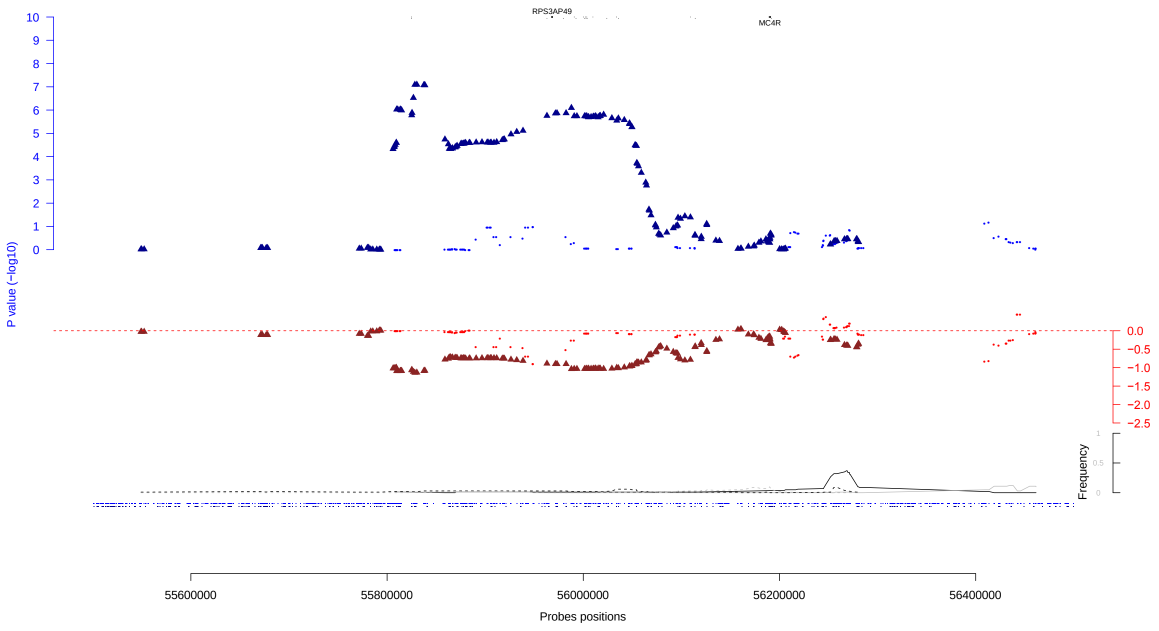
RPS3AP49
MC4R
P value (−log10)
Frequency
Probes positions
55600000
55800000
56000000
56200000
56400000

B

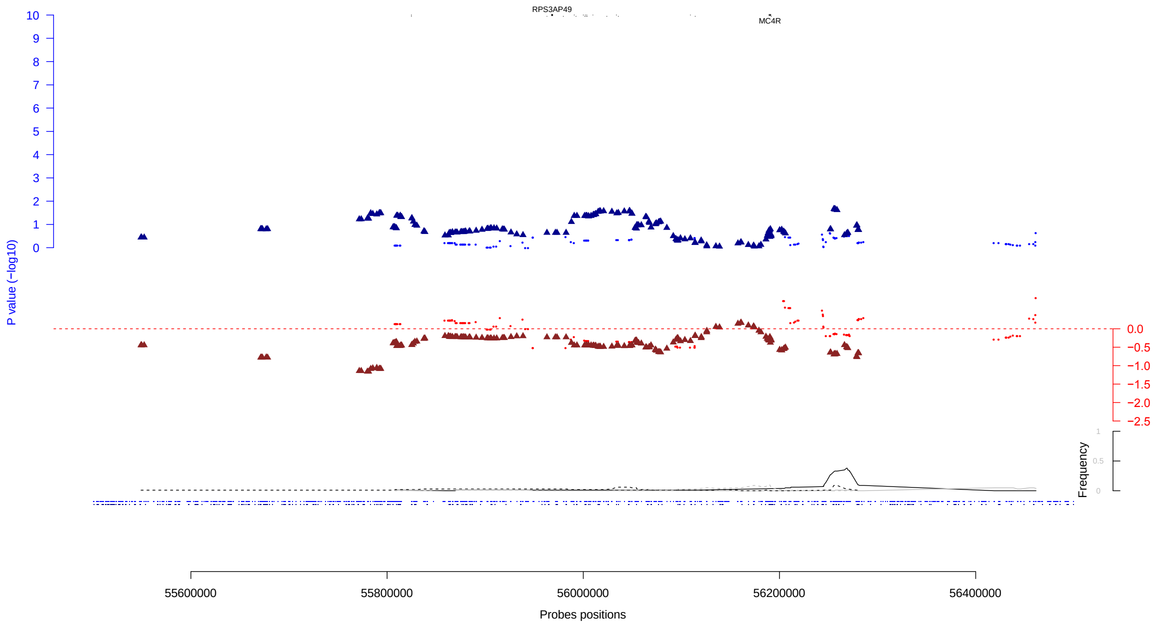
RPS3AP49
MC4R
P value (−log10)
Frequency
Probes positions
55600000
55800000
56000000
56200000
56400000

C

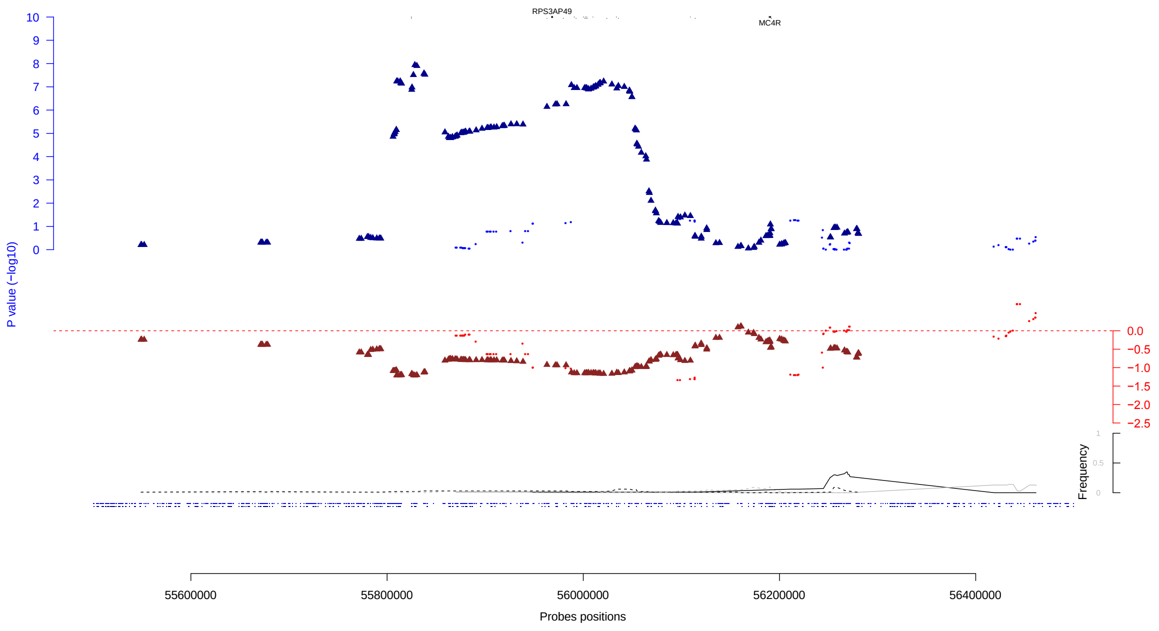

Supplementary Figure 10: (A) Local Manhattan plot on the rearrangement near MC4R, separately for the GIANT data (dot) and the UKBB data (triangles). The blue dots/triangles correspond to the probes p-values of the association with the BMI. The red dots/triangles are the corresponding effect sizes. At the bottom the black and grey lines are the deletions and duplications frequencies. The plain one corresponds to the GIANT data while the dash one to the UKBB. Finally, the dots at the bottom are the probes positions for the GIANT cohorts above and the UK Biobank below. The position of the MC4R gene is set at the top of the plot with the position of the BMI associated probes from GWAS. **(B)** Local Manhattan plot on the rearrangement near MC4R, separately for the GIANT data (dot) and the UKBB data (triangles). The blue dots/triangles correspond to the probes p-values of the association with the Height. The red dots/triangles are the corresponding effect sizes. At the bottom the black and grey lines are the deletions and duplications frequencies. The plain one corresponds to the GIANT data while the dash one to the UKBB. Finally, the dots at the bottom are the probes positions for the GIANT cohorts above and the UK Biobank below. The position of the MC4R gene is set at the top of the plot with the position of the BMI associated probes from GWAS. (**C)** Local Manhattan plot on the rearrangement near MC4R, separately for the GIANT data (dot) and the UKBB data (triangles). The blue dots/triangles correspond to the probes p-values of the association with the Weight. The red dots/triangles are the corresponding effect sizes. At the bottom the black and grey lines are the deletions and duplications frequencies. The plain one corresponds to the GIANT data while the dash one to the UKBB. Finally, the dots at the bottom are the probes positions for

the GIANT cohorts above and the UK Biobank below. The position of the MC4R gene is set at the top of the plot with the position of the BMI associated probes from GWAS.

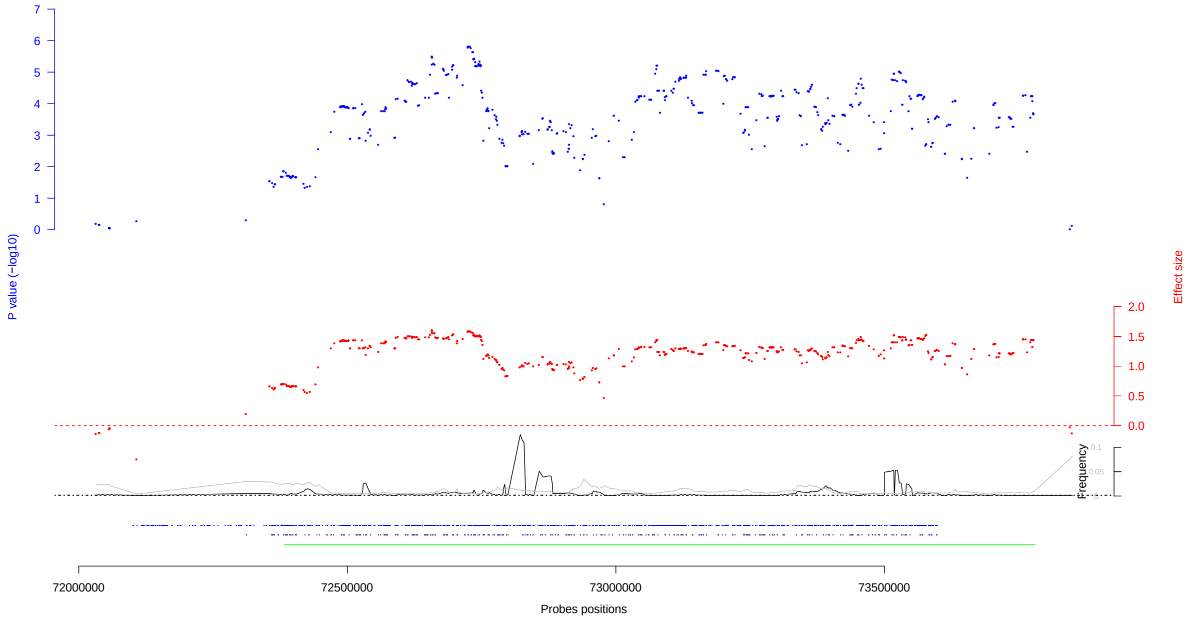

Supplementary Figure 11: Local Manhattan plot on the 7q11.23 rearrangement. The blue dots correspond to the probes p-values of the association with the Waist-Hip ratio. The red dots are the corresponding effect sizes. At the bottom the black and grey lines are the deletions and duplications frequencies. Finally, the dots at the bottom are the probes positions for the GIANT cohorts above and the UK Biobank below. The green line represents the known syndromic CNV in the region.

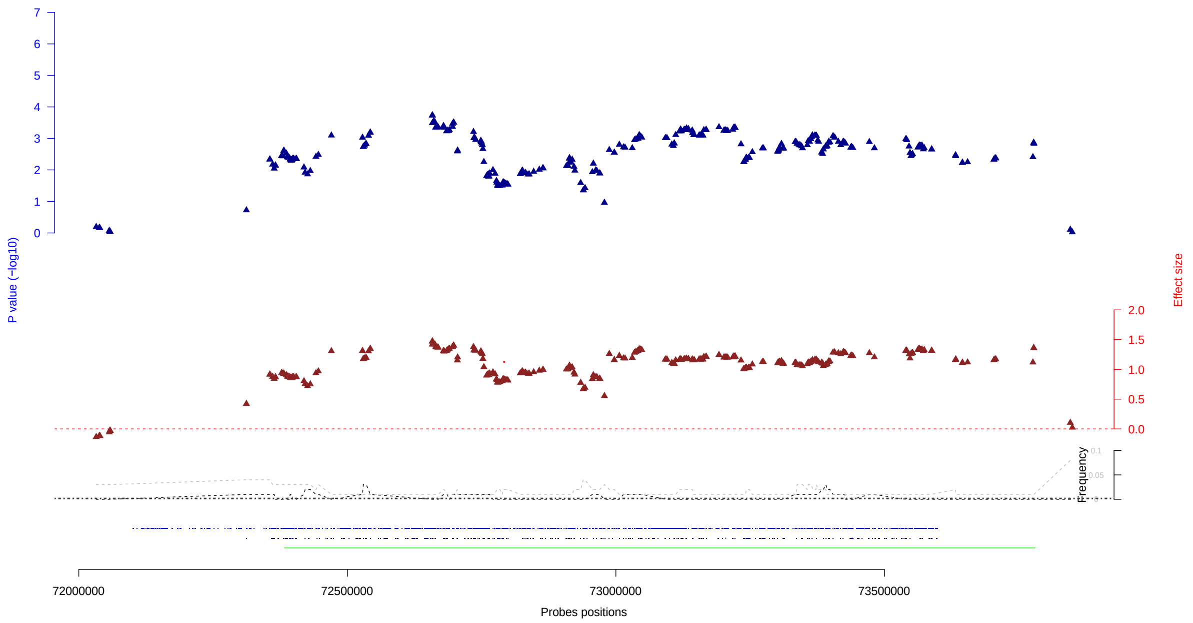

Supplementary Figure 12: Local Manhattan plot on the 7q11.23 rearrangement, separately for the GIANT data (dot) and the UKBB data (triangles). The blue dots/triangles correspond to the probes p-values of the association with the Waist-Hip ratio. The red dots/triangles are the corresponding effect sizes. At the bottom the black and grey lines are the deletions and duplications frequencies. The plain one corresponds to the GIANT data while the dash one to the UKBB. Finally, the dots at the bottom are the probes positions for the GIANT cohorts above and the UK Biobank below. The green line represents the known syndromic CNV in the region.

A

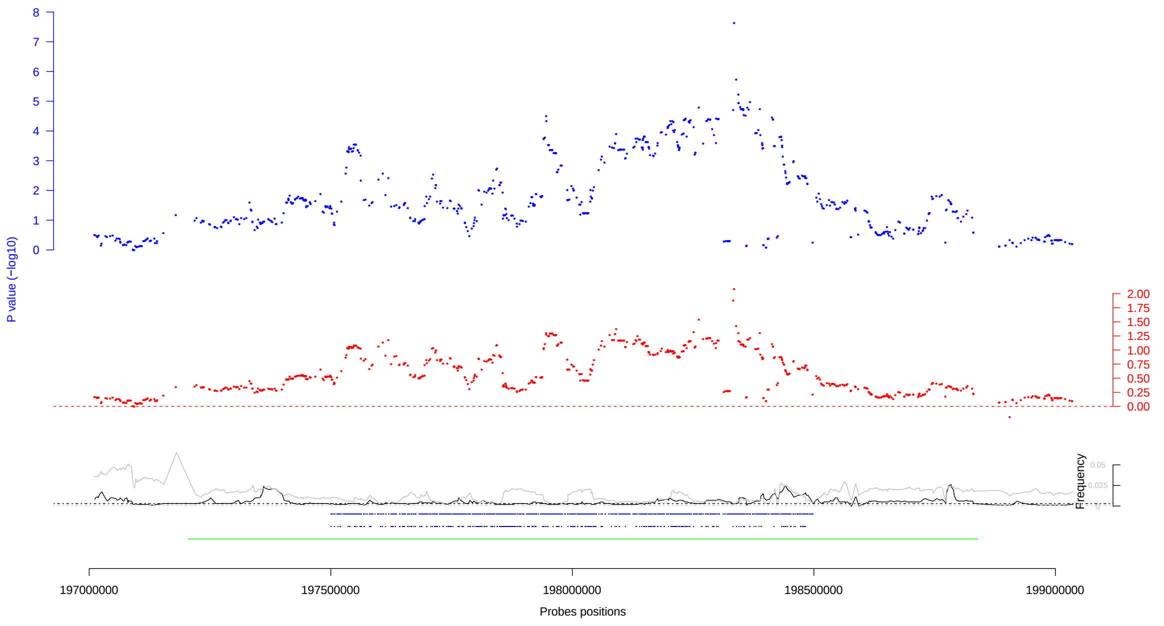

B

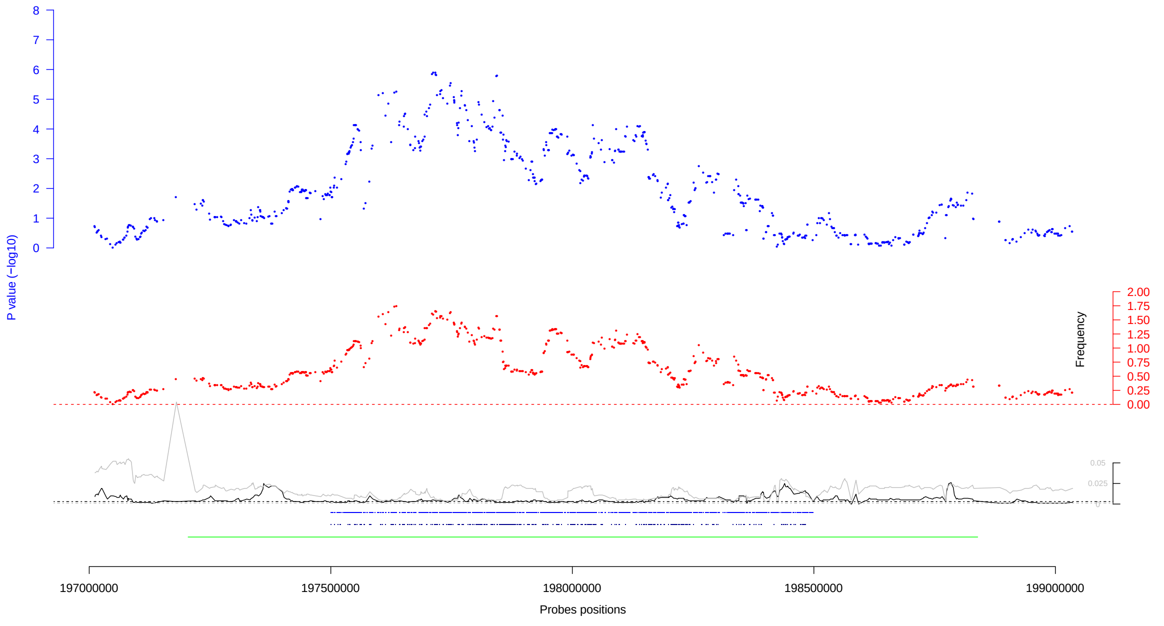

Supplementary Figure 13: (A) Local Manhattan plot on the 3q29 rearrangement. The blue dots correspond to the probes p-values of the association with the Height. The red dots are the corresponding effect sizes. At the bottom the black and grey lines are the deletions and duplications frequencies. Finally, the dots at the bottom are the probes positions for the GIANT cohorts above and the UK Biobank below. The green line represents the known

syndromic CNV in the region. (**B)** Local Manhattan plot on the 3q29 rearrangement. The blue dots correspond to the probes p-values of the association with the Weight. The red dots are the corresponding effect sizes. At the bottom the black and grey lines are the deletions and duplications frequencies. Finally, the dots at the bottom are the probes positions for the GIANT cohorts above and the UK Biobank below. The green line represents the known syndromic CNV in the region.

A

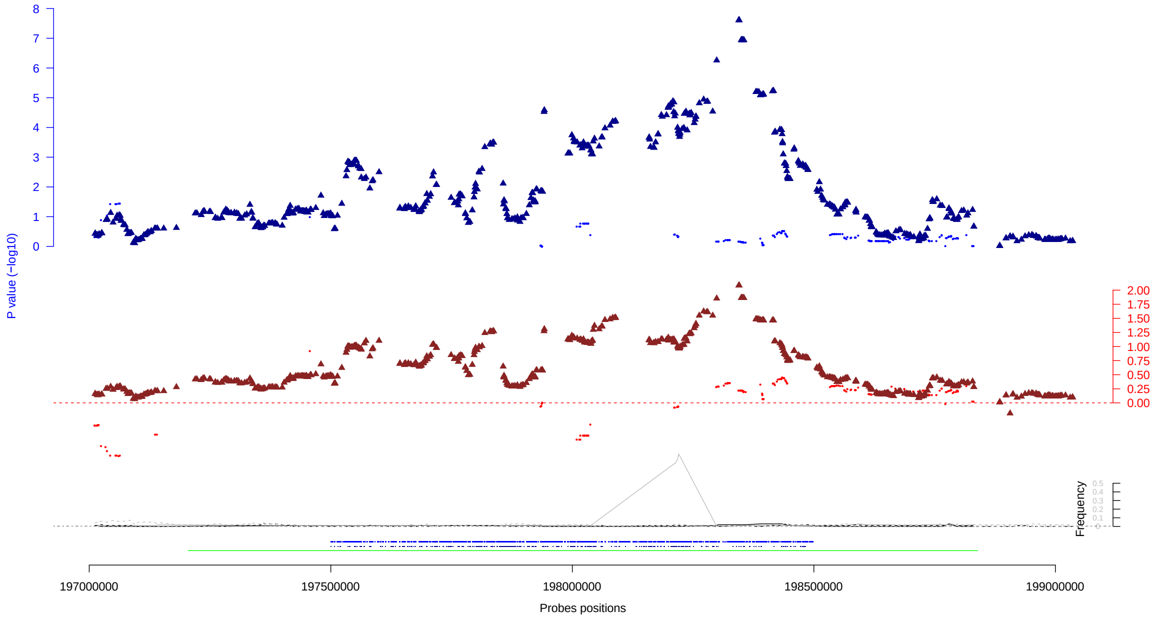

B

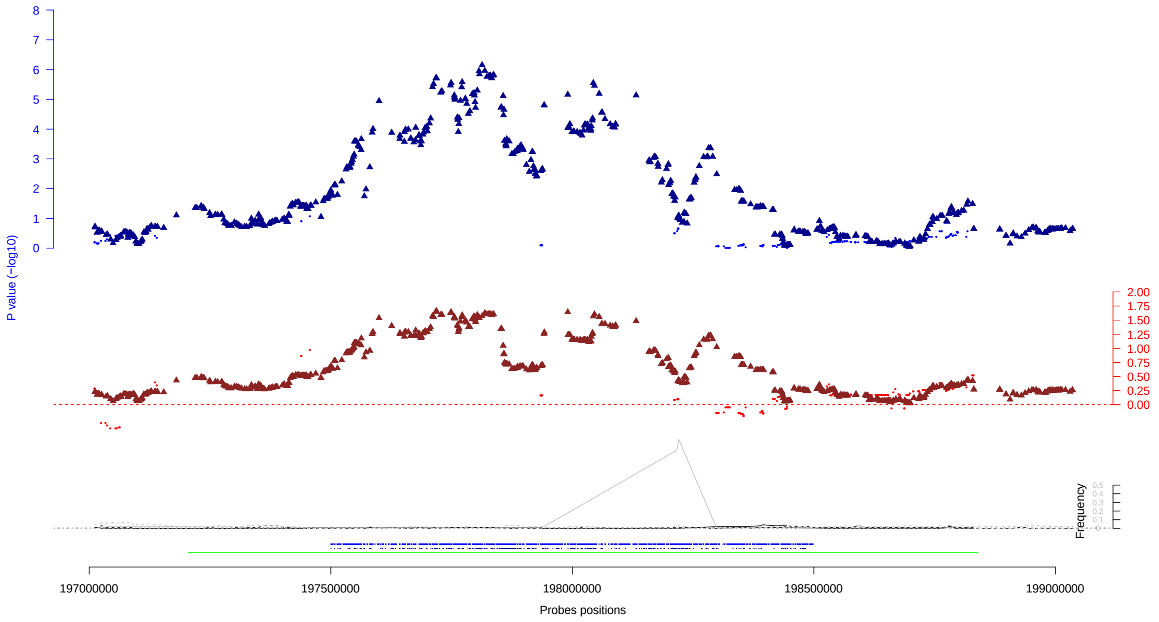

Supplementary Figure 14: (A) Local Manhattan plot on the 3q29 rearrangement, separately for the GIANT data (dot) and the UKBB data (triangles). The blue dots/triangles correspond to the probes p-values of the association with the Height. The red dots/triangles are the corresponding effect sizes. At the bottom the black and grey lines are the deletions and duplications frequencies. The plain one corresponds to the GIANT data while the dash one

to the UKBB. Finally, the dots at the bottom are the probes positions for the GIANT cohorts above and the UK Biobank below. The green line represents the known syndromic CNV in the region. (**B)** Local Manhattan plot on the 3q29 rearrangement, separately for the GIANT data (dot) and the UKBB data (triangles). The blue dots/triangles correspond to the probes p-values of the association with the Weight. The red dots/triangles are the corresponding effect sizes. At the bottom the black and grey lines are the deletions and duplications frequencies. The plain one corresponds to the GIANT data while the dash one to the UKBB. Finally, the dots at the bottom are the probes positions for the GIANT cohorts above and the UK Biobank below. The green line represents the known syndromic CNV in the region.

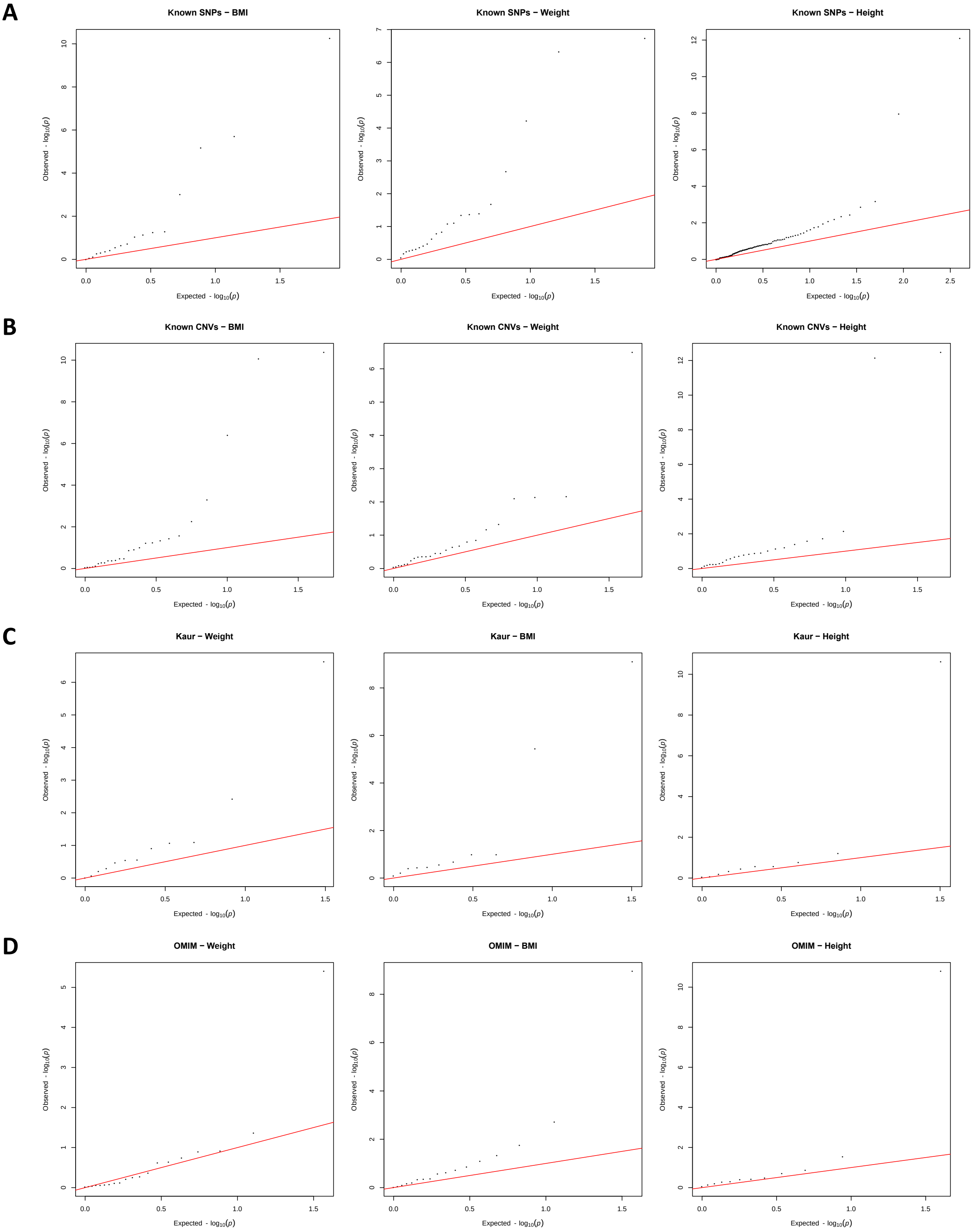

A
Known SNPs − BMI
Known SNPs − Weight
Known SNPs − Height
B
Known CNVs − BMI
Known CNVs − Weight
Known CNVs − Height
C
Kaur − Weight
Kaur − BMI
Kaur − Height
D
OMIM − Weight
OMIM − BMI
OMIM − Height
Observed - log10(p)
Expected - log10(p)

Supplementary Figure 15: Q-Q plots for the association between probes falling in various candidate regions. The columns correspond to the traits of interest: BMI (left), weight (middle) and height (right). **(A)** Regions are selected to be within 500kb vicinity of SNPs associated with BMI[1] or height[2]. For the 97 and 423 candidate regions for BMI and height, respectively, we did not observe significant enrichment of CNVs with *minP*<0.05 (Supplementary Table 24). Beyond the already known 16p11.2 CNV, the *MC4R* CNVs survived multiple testing correction for BMI and the 1q21.1 CNV (close to the height SNP rs6658763 in gene *FMO5*) for height. Note however that both were picked up in the genome-wide scan and hence described in detail in the main text. **(B)** Regions selected based on 59 previously BMI-associated CNVs (Peterson et al. [3]), out of which we tested 39 that were covered by probes with high quality CNV calls (r^2>0.5). For all probes within these CNVs we collected the association p-values for BMI. For each of these CNVs we then calculated the minimal association P-value in our study for BMI and corrected them for the number of encompassed independent probes in the given CNV, denoting it with *minP*. We observed four CNVs with *minP*<0.05, corresponding to 2.1-fold enrichment (P=0.04). Only the 16p11.2 CNVs survive multiple testing correction (minP<0.05/39) (Supplementary Table 25). **(C)** We repeated the experiment with a newly proposed set of genes shown to be involved in some genetic syndrome with obesity[4]. Out of the testable 65 unique candidate genes 57 are located on autosomes, among these 54 had copy number variant probes, 32 of which with reasonable imputation quality (r^2>0.5). We observed a non-significant 1.2-fold enrichment of candidates with minP<0.05 (P=0.21). Note that 23 of the 32 genes had corrected P-value equal to 1, thus only 10 distinct points are visible. Only the 16p11.2 CNV survived multiple testing correction. **(D)** Finally, we looked at regions harbouring genes related to syndromic height and or weight alterations according to the OMIM repository[5] (selection made on height-, weight- and BMI-related key words). We observed a slight inflation on the QQ-plots for the 37 BMI / weight and 40 height candidates and found 2.2-fold enrichment of candidates with minP<0.05 for BMI (P=0.04). Here again, only the 16p11.2 CNV survived multiple testing correction.

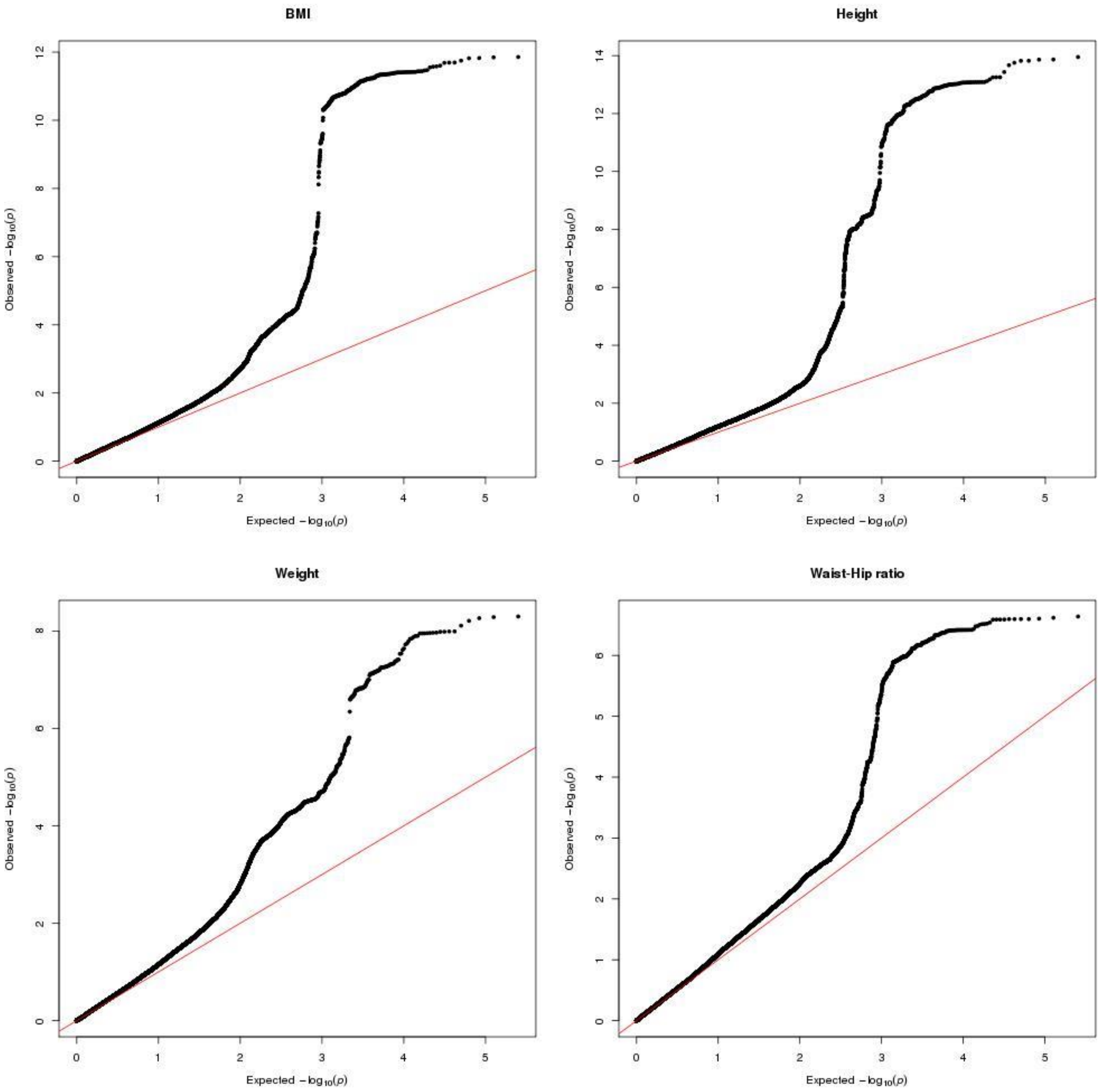

Supplementary Figure 16: Quantile-quantile plots of the copy number association P-values for BMI, height, weight and waist-to-hip ratio.

Supplementary Tables

	Beta				P-value			
	All mirror	del	dup	U-shaped	All mirror	del	dup	U-shaped
BMI	3.33E-02	1.44E-01	3.15E-02	4.98E-02	3.74E-02	2.85E-03	1.40E-01	1.06E-02
Weight	2.35E-02	1.12E-01	1.12E-02	2.73E-02	6.22E-01	4.46E-01	8.61E-01	6.42E-01
Height	-8.37E-02	-4.13E-01	-7.84E-02	-1.32E-01	8.56E-05	2.51E-10	6.00E-03	4.46E-07
WHR	6.93E-04	2.94E-03	8.21E-04	1.15E-03	3.37E-03	6.03E-05	9.18E-03	6.90E-05

Supplementary Table 1: CNV burden analysis results per trait and CNV categories.

chr	start	end	Beta BMI			P val BMI			R square BMI			Beta WT			P val WT		
			mirror	del	dup	mirror	del	dup	mirror	del	dup	mirror	del	dup	mirror	del	dup
1	145	145.9	0.92	-1.11	1.37	3.97E-02	1.41E-01	1.78E-02	0.80	0.77	0.50	6.66	-9.66	6.27	1.73E-06	1.52E-05	4.83E-04
3	197.7	197.9	5.00		7.2	1.55E-03		6.17E-04	0.98		0.64	22.55		24.14	1.59E-06		1.20E-04
3	198.2	198.4	2.93		2.71	3.53E-02		1.89E-01	1.00		0.65	15.88	-11.68	11.49	1.22E-04	5.13E-02	6.18E-02
7	72.66	72.75	4.59			4.40E-03			0.97			14.41			2.60E-03		
11	26.97	27.19	1.46	-0.56	2.01	5.89E-02	1.55E-01	1.15E-01	0.86	0.92	0.96	2.82	-3.09	3.02	1.09E-02	9.52E-03	4.55E-01
16	28.73	28.95	-3.07	5.16	-2.08	5.31E-08	5.51E-06	4.44E-03	0.76	0.78	0.59	-10.35	14.57	-8.01	5.03E-09	1.44E-05	3.46E-04
16	29.5	30.11	-3.66	6.15	-1.81	1.39E-12	4.48E-14	1.16E-02	1.00	0.99	0.77	-6.57	12.5	-2.52	5.25E-05	1.79E-06	2.83E-01
18	55.81	56.05	-5.06	5.66	2.22	2.03E-07	5.75E-08	2.24E-01	0.92	0.92	0.60	-15.94	17.43	3.77	1.45E-08	2.16E-08	5.27E-01
Close to significance CNVs																	
2	111.3	111.5	-2.30	2.54	-2.22	6.23E-04	1.38E-02	1.88E-02	1.00	1.00	1.00	-8.97	7.11	-9.98	1.33E-05	2.52E-02	2.82E-04
2	112.4	112.5	-2.15	2.19	-2.03	4.96E-04	4.07E-02	8.15E-03	0.91	0.91	0.91	-8.77	7.64	-8.43	1.74E-05	3.26E-02	2.68E-04
15	20.33	20.62	-0.22	0.36	-1.52	2.78E-01	2.97E-01	9.93E-02	0.94	0.96	0.88	1.2	-2.18	0.48	5.88E-03	2.55E-03	3.75E-01
22	17.41	18.69	2.11	4	2.05	1.70E-05	1.54E-02	4.43E-06	0.96	0.97	0.94	5.01	6.12	5.09	5.83E-04	2.05E-01	1.52E-03
22	19.07	19.33	1.85	-1.71	1.89	3.82E-06	1.80E-01	1.40E-05	1.00	1.00	1.00	4.98	-8.42	5.28	5.26E-05	9.59E-02	5.95E-05

chr	start	end	Beta HT			P val HT			R square HT			Beta WHR			P val WHR		
			mirror	del	dup	mirror	del	dup	mirror	del	dup	mirror	del	dup	mirror	del	dup
1	145	145.9	3.46	-6.57	2.22	3.75E-10	3.30E-11	1.27E-03	0.93	0.95	0.89	0.020	-0.010	0.028	2.24E-03	3.02E-01	1.46E-03
3	197.7	197.9	8.29		1.44	3.12E-05		3.95E-01	0.96		0.93	0.028	0.060	0.095	3.03E-02	1.35E-01	4.50E-03
3	198.2	198.4	13.3			2.32E-08			0.95			-0.046	0.061	0.067	8.07E-03	2.16E-02	2.88E-02
7	72.66	72.75	3.14			1.91E-01			0.73			0.111	-0.153	0.104	1.49E-06	2.75E-02	8.43E-05
11	26.97	27.19	2.43	-2.56	-2.64	1.46E-06	8.24E-08	1.24E-01	0.95	0.82	0.89	0.022	-0.014	-0.053	5.69E-02	2.11E-01	7.41E-02
16	28.73	28.95	-1.6	-0.62	-2.81	4.24E-02	6.79E-01	5.29E-03	0.97	0.96	0.97	-0.024	0.057	-0.037	1.25E-02	8.67E-04	2.20E-01
16	29.5	30.11	5.21	-8.32	3.43	1.12E-14	5.44E-14	3.04E-04	0.99	0.98	0.97	-0.041	0.092	-0.057	2.30E-07	2.20E-11	3.02E-02
18	55.81	56.05	-3.16	2.87	-2.93	1.41E-02	4.20E-02	2.37E-01	0.97	0.97	0.64	-0.036	0.035	-0.059	8.86E-03	2.41E-02	2.15E-01
Close to significance CNVs																	
2	111.3	111.5	-2.74	-0.81	-4.06	2.26E-03	5.73E-01	4.86E-04	0.99	1.00	0.98	-0.015	0.032	0.032	1.61E-01	6.00E-02	3.90E-01
2	112.4	112.5	-2.18	-0.99	-3.21	7.89E-03	4.93E-01	1.61E-03	0.88	1.00	0.98	-0.014	0.028	-0.011	1.52E-01	1.33E-01	3.55E-01
15	20.33	20.62	0.74	-1.49	0.38	8.10E-06	3.14E-08	1.28E-01	0.95	0.94	0.95	0.002	0.006	0.007	3.48E-01	3.16E-02	1.08E-02
22	17.41	18.69	-1.36	-6.76	-1.84	2.89E-02	1.37E-03	5.64E-03	0.97	0.96	1.00	0.027	0.067	0.022	3.11E-03	8.13E-03	9.17E-04
22	19.07	19.33	-0.62	-2.44	-1.99	2.94E-01	1.63E-01	2.49E-01	1.00	1.00	0.58	0.020	0.041	0.022	1.55E-03	1.96E-01	4.58E-04

Supplementary Table 2: CNV association meta-analysis results considering mirror, only deletion and only duplication effects models. (Human genome build 36)

Chr	Start (Mb)	End (Mb)	Genes	OMIM diseases
1	145	145.9	RN7SL261P RNVU1-8 CHD1L NBPF13P GJA8 OR13Z3P LINC00624 OR13Z2P OR13Z1P PDIA3P1 FMO5 RPL7AP15 CCT8P1 PRKAB2 GJA5 GPR89B BCL9 ACP6	Atrial_fibrillation,_familial,_11,_614049_(3),_Autosomal_dominant<br>Atrial_standstill,_digenic_(GJA5/SCN5A),_108770_(3),_Autosomal_dominant<br>Cataract_1,_multiple_types,_116200_(3),_Autosomal_dominant
16	28.73	28.95	MIR4721 MIR4517 ATXN2L SH2B1 CD19 RABEP2 TUFM ATP2A1 NFATC2IP ATP2A1-AS1 LAT SPNS1	Combined_oxidative_phosphorylation_deficiency_4,_610678_(3)<br>Brody_myopathy,_601003_(3),_Autosomal_recessive<br>Immunodeficiency,_common_variable,_3,_613493_(3),_Autosomal_recessive
16	29.5	30.1	MIR3680-2 RN7SKP127 C16orf54 PAGR1 CORO1A MAZ ALDOA CDIPT MVP ZG16 SEZ6L2 CDIPT-AS1 PRRT2 YPEL3 TMEM219 DOC2A GDPD3 INO80E KCTD13 HIRIP3 ASPHD1 MAPK3 TAOK2 PPP4C FAM57B C16orf92 SMG1P2 SLC7A5P1 CA5AP1 QPRT SPN TBX6 KIF22	Spondyloepimetaphyseal_dysplasia_with_joint_laxity,_type_2,_603546_(3),_Autosomal_dominant<br>Convulsions,_familial_infantile,_with_paroxysmal_choreoathetosis,_602066_(3),_Autosomal_dominant<br>_Episodic_kinesigenic_dyskinesia_1,_128200_(3),_Autosomal_dominant<br>_Seizures,_benign_familial_infantile,_2,_605751_(3),_Autosomal_dominant<br>Glycogen_storage_disease_XII,_611881_(3),_Autosomal_recessive<br>Spondylocostal_dysostosis_5,_122600_(3),_Autosomal_dominant<br>Immunodeficiency_8,_615401_(3),_Autosomal_recessive

Supplementary Table 3: List of OMIM diseases for the genes located in the discovered CNVs. Only three CNVs contained genes related to OMIM diseases. (human genome build 36)

Pheno	Beta (Inverse Norm)	P
Reaction Time when Paying SNAP	1.00317961301502	5.81999E-05
Waist Circumference	1.37197342090424	0.000113284
BMI	1.31488002324912	0.000216001
Basal Metabolic Rate	1.28061436732642	0.000314676
Townsend Deprivation Index	0.824668449619557	0.000521569
Mental Health Score	2.27456836098822	0.001463805
Hearing Right	2.9938759978349	0.00171535
Income Category	-0.836122729862511	0.00184698
Hand Grip Strength	-1.76087217905607	0.002472095
Height	1.34326187246243	0.002747451
Forced Vital Capacity	-1.09919898286292	0.003410296
Bodyfat Percentage	1.00060529716209	0.00487609
Fluid Intelligence Test Score	-1.45747890521265	0.013103228
Number of Depression Episodes	5.44797580489105	0.013407919
Age At Menarche	-1.37000661836451	0.014310623
Bone Mineral Density	12.421391675403	0.016604821
Forced Expiratory Volume	-0.566226688699171	0.02301327
Diastolic Blood Pressure	-0.531416055206	0.039000511
Age when left higher education	-13.7931769953107	0.084191421
Number of Hours Slept Per Night	-0.445253506751197	0.098055243
WHR corrected for BMI	-0.416673511307246	0.130104338
Number of Government Health Advice Ignored	8.41028932318149	0.139300258
Hearing Left	-0.706324244835924	0.175876902
Pairs Matching Test Score	0.327003334128527	0.177431468
Birthweight	0.544289288478213	0.19444689
General Happiness Score	0.578526516814893	0.267346397
Systolic Blood Pressure	0.631905434350093	0.276574468

Supplementary Table 4: Top UK Biobank traits association – 16p11.2 220kb Deletion

Pheno	Beta (Inverse Norm)	P
Forced Expiratory Volume	-1.16665918180088	9.96653E-07
Hand Grip Strength	-1.42085821243649	2.75524E-05
Forced Vital Capacity	-1.51127055927164	0.000135717
Income Category	-0.636507603844169	0.000226834
Age when left higher education	-0.724872128280916	0.000521376
Reaction Time when Paying SNAP	0.78068371874827	0.000572615
Townsend Deprivation Index	0.716772068895736	0.001019951
Systolic Blood Pressure	1.20115838609033	0.006725133
Birthweight	0.63769736386775	0.006826511
Fluid Intelligence Test Score	-0.78626798087886	0.006903458
Hearing Right	1.50223519328564	0.008576829
Basal Metabolic Rate	-0.412179984464185	0.010134824
BMI	-0.400806059955445	0.011658998
Bone Mineral Density	-0.40673802129927	0.012341872
Bodyfat Percentage	-0.527408770942645	0.019984666
Number of Government Health Advice Ignored	0.691382076183591	0.020682411
Age At Menarche	0.530042233521143	0.028951357
Diastolic Blood Pressure	0.946819872774768	0.032662582
Number of Depression Episodes	-2.10459935533373	0.039050899
Height	0.968071430550906	0.039216385
Mental Health Score	0.3603553649529	0.040528695
Pairs Matching Test Score	1.9813718334488	0.048629148
Waist Circumference	-0.301237950238058	0.057804952
WHR corrected for BMI	-0.578839011164971	0.063129236
Hearing Left	0.771186459244182	0.081212421
Number of Hours Slept Per Night	1.72857449125901	0.085393952
General Happiness Score	0.817177716204734	0.122648178

Supplementary Table 5: Top UK Biobank traits association – 16p11.2 220kb Duplication

Pheno	Beta (Inverse Norm)	P
Bodyfat Percentage	1.67602288645414	2.85152E-13
Waist Circumference	1.59138567876272	3.47323E-13
BMI	1.507991093061	5.45371E-12
Forced Vital Capacity	-1.71837638025093	6.80729E-12
Townsend Deprivation Index	1.3519201060548	1.46053E-11
Height	-1.44694062381608	1.02805E-10
Income Category	-1.48617024171029	4.42269E-10
Forced Expiratory Volume	-1.52718398860764	1.07274E-09
Hand Grip Strength	-1.25366176070497	1.3952E-09
Birthweight	-24.413562402529	0.000663086
Pairs Matching Test Score	3.26474311293541	0.001154876
Age At Menarche	-1.08838702695159	0.001248673
Mental Health Score	-1.18006009631771	0.001997616
Hearing Right	1.51080468265243	0.002232018
Reaction Time when Paying SNAP	0.76851872869137	0.002295937
Bone Mineral Density	0.863093422674174	0.002482608
Number of Government Health Advice Ignored	0.899851301036471	0.003524589
Number of Depression Episodes	15.5668732945112	0.004996177
Hearing Left	1.15567077893393	0.005205249
Basal Metabolic Rate	0.835131109211715	0.012296668
Fluid Intelligence Test Score	-1.28637989085312	0.016493523
Systolic Blood Pressure	0.598220369311587	0.017624569
WHR corrected for BMI	2.27147205457894	0.023758019
Diastolic Blood Pressure	0.915990420047258	0.026426458
Number of Hours Slept Per Night	1.26593940462379	0.029168054
Age when left higher education	-0.427704313588078	0.074265546
General Happiness Score	1.17321874481301	0.103167054

Supplementary Table 6: Top UK Biobank traits association – 16p11.2 600kb Deletion

Pheno	Beta (Inverse Norm)	P
Bone Mineral Density	-0.740567984627244	1.21264E-05
Reaction Time when Paying SNAP	0.768538430108658	2.20774E-05
Mental Health Score	1.50395410772168	6.50798E-05
Age when left higher education	2.01516893752163	0.000542613
Fluid Intelligence Test Score	-1.42401828491299	0.000626779
Townsend Deprivation Index	1.22458374660638	0.001135683
Income Category	-0.782895517463454	0.001559736
Systolic Blood Pressure	-0.668623806892171	0.002253388
Hand Grip Strength	-0.585129887482488	0.002258687
Height	0.540983456555768	0.002392488
Age At Menarche	0.683432373697753	0.002859066
General Happiness Score	0.901502943851121	0.004052996
Diastolic Blood Pressure	-0.619807879304054	0.004632047
Number of Depression Episodes	1.29717891918402	0.00562968
Forced Vital Capacity	-0.734918377470063	0.006426071
Forced Expiratory Volume	-0.730805045822161	0.006729204
Bodyfat Percentage	-0.455881499473242	0.007913018
BMI	-0.545792509228783	0.008953892
WHR corrected for BMI	1.00287662493425	0.014731776
Birthweight	0.599392287080348	0.017546555
Number of Government Health Advice Ignored	1.63368521958997	0.020999733
Waist Circumference	-1.12949043737894	0.026039595
Basal Metabolic Rate	-1.26949880676746	0.030306993
Number of Hours Slept Per Night	1.39967987876525	0.049720071
Pairs Matching Test Score	0.800198859677159	0.053917198
Hearing Right	-15.0929064726569	0.06068741
Hearing Left	1.20560783138371	0.234714015

Supplementary Table 7: Top UK Biobank traits association – 16p11.2 600kb Duplication

Chr	Start (Mb)	End (Mb)	BMI			Height		
			SNP	Nearest gene	P-value	SNP	Nearest gene	P-value
1	145	145.9	-	-	-	rs6658763	FMO5	3.60E-11
16	28.73	28.95	rs3888190	ATP2A1	3.14E-23	-	-	-
16	29.5	30.1	-	-	-	rs11642612	FLJ25404	4.20E-08
18	55.81	56.05	rs6567160	MC4R	3.93E-53	rs11152213	MC4R	6.90E-13

Supplementary Table 8: Known SNPs (from previous BMI and height GWAS) and their nearest gene located in the discovered CNV. (Human genome build 36)

SNP	Allele	Gene	SNP - Height		SNP - Expression		CNV - Height	
			Beta	P-value	Z score	P-value	Beta	P-value
rs11150581	C	CORO1A	-0.017	4.29E-08	-4.45	8.60E-06	0.82	1.10E-14
rs11642612	C	CORO1A	-0.018	1.24E-08	-4.45	8.60E-06	0.82	1.10E-14
rs11150581	C	INO80E	-0.017	4.29E-08	-5.57	2.55E-08	0.82	1.10E-14
rs11642612	C	INO80E	-0.018	1.24E-08	-5.57	2.55E-08	0.82	1.10E-14
rs2278557	G	INO80E	-0.017	4.64E-08	-5.52	3.49E-08	0.82	1.10E-14
rs6565173	G	INO80E	0.016	6.68E-08	5.8	6.54E-09	0.82	1.10E-14
rs9925915	G	INO80E	0.016	8.97E-08	5.8	6.48E-09	0.82	1.10E-14

Supplementary Table 9: eQTLs SNPs located in the discovered CNVs and their effect on the gene expression and the trait of interest (height).

Pheno	Beta (Inverse Norm)	P
Height	-1.19391234559082	1.35706E-11
Basal Metabolic Rate	-1.10744807923833	8.64828E-08
Forced Expiratory Volume	-0.928102424720159	2.26727E-07
Forced Vital Capacity	-0.919381302886394	2.93872E-07
Hand Grip Strength	-0.678840894146804	1.05726E-05
Income Category	-0.797375544388421	0.000164561
Age At Menarche	-2.18111621831189	0.000181161
WHR corrected for BMI	1.21135856746827	0.001584752
Birthweight	-1.47199099115565	0.003644883
Pairs Matching Test Score	0.616587892276118	0.004196544
Hearing Left	-1.42549588498061	0.005746628
Waist Circumference	-0.534637932226143	0.008312489
Townsend Deprivation Index	0.386471692056822	0.008422622
Bone Mineral Density	-0.510569581710803	0.010557709
BMI	0.663142062152245	0.014348222
Reaction Time when Paying SNAP	0.383757729096462	0.01492828
Age when left higher education	-0.513806000930208	0.015202114
Bodyfat Percentage	-0.483667006516757	0.019393833
Number of Depression Episodes	-67.4062302105066	0.021836674
Systolic Blood Pressure	-0.319695603480369	0.024014062
Number of Hours Slept Per Night	-0.774320279114834	0.041964854
Mental Health Score	0.33584883482338	0.053873586
Diastolic Blood Pressure	-0.268899170395633	0.067343385
Hearing Right	-1.27529914599644	0.087466989
General Happiness Score	-1.68598097949115	0.123367387
Fluid Intelligence Test Score	0.492888873070761	0.400399548
Number of Government Health Advice Ignored	0.224845167635341	0.403158358

Supplementary Table 10: Top UK Biobank traits association – 1q21.1 Deletion

Pheno	Beta (Inverse Norm)	P
Bodyfat Percentage	0.51654168843782	9.08923E-05
Forced Expiratory Volume	-0.683510779833834	0.000115131
Townsend Deprivation Index	1.19138021400774	0.000285928
Income Category	-0.614381117971488	0.000300941
Height	0.441894094239297	0.000541124
Waist Circumference	0.474845948716028	0.000615927
Number of Depression Episodes	-3.2983860848696	0.0014689
Fluid Intelligence Test Score	-0.713141844035933	0.001895973
General Happiness Score	0.629481571940363	0.00329901
Reaction Time when Paying SNAP	0.381771713057794	0.004356698
Basal Metabolic Rate	0.358242439834419	0.005118429
Age At Menarche	1.62211117592782	0.005599322
Forced Vital Capacity	-0.392373795751726	0.006215335
BMI	0.326494280777879	0.014220087
Hand Grip Strength	-0.311609113952823	0.019090265
Birthweight	0.511073938454779	0.019203328
Number of Hours Slept Per Night	0.904682488433287	0.021581146
Pairs Matching Test Score	0.71928863958202	0.028514502
Number of Government Health Advice Ignored	0.375950761958496	0.030303827
Age when left higher education	-0.357327073405086	0.039093313
Mental Health Score	0.303323689959939	0.046395222
Hearing Left	0.417263493960358	0.060192985
Systolic Blood Pressure	0.837225651515229	0.076127705
Diastolic Blood Pressure	0.25130483232228	0.086317433
Bone Mineral Density	0.213679010137385	0.122432571
Hearing Right	0.710200126988719	0.125452515
WHR corrected for BMI	-0.180971453265608	0.160369302

Supplementary Table 11: Top UK Biobank traits association – 1q21.1 Duplication

Chr	Start (Mb)	End (Mb)	Genes	CNV beta	CNV – P-value	SNP	Nearest gene	SNP – P-value
11	26.97	27.19	FIBIN – BBOX1	0.38	1.50E-06	rs138273386	FIBIN	5.79E-12

Supplementary Table 12: Exome SNPs associated with height. The SNP comes from a GIANT-exome study on height (Marouli et al. 2017) including 381,625 individuals. (Human genome build 36)

Pheno	Beta (Inverse Norm)	P
Bone Mineral Density	2.5937508362453	0.009804329
Townsend Deprivation Index	-1.14323622381117	0.011617731
Age when left higher education	-0.942196492847463	0.038182179
Hearing Right	-1.58214258999308	0.044129454
Reaction Time when Paying SNAP	-0.596752588357782	0.117216709
Hand Grip Strength	0.551083090367888	0.126981314
Age At Menarche	-0.774216818738854	0.138082171
General Happiness Score	42.9671566783249	0.165456672
Height	-1.35358929756997	0.177712004
Income Category	-0.77680692429136	0.185088056
Pairs Matching Test Score	-1.303907968215	0.194157299
Birthweight	-1.30762895069462	0.194503651
Number of Depression Episodes	1.51147620435941	0.216405206
Systolic Blood Pressure	-0.460878088491214	0.225718408
Basal Metabolic Rate	0.537022600631399	0.235915959
BMI	1.18694196140479	0.237252814
Bodyfat Percentage	1.1623343872493	0.247135329
Fluid Intelligence Test Score	33.367868574876	0.281619632
Forced Expiratory Volume	-0.427819714080169	0.300924933
Mental Health Score	-0.999879807230626	0.319716683
Diastolic Blood Pressure	-0.377313002867347	0.321295215
WHR corrected for BMI	-0.355653121189768	0.389310308
Waist Circumference	0.853417840972687	0.395436814
Hearing Left	0.54223103946467	0.490424197
Number of Hours Slept Per Night	-0.376099142160308	0.520786991
Forced Vital Capacity	0.3720465161274	0.525652856
Number of Government Health Advice Ignored	-0.311022413453132	0.536978353

Supplementary Table 13: Top UK Biobank traits association – *FIBIN BBOX1* Deletion

Pheno	Beta (Inverse Norm)	P
Bone Mineral Density	-1.2518564188802	0.004182866
Townsend Deprivation Index	1.00253197304022	0.020692661
General Happiness Score	-2.67385086771405	0.025387429
Hearing Left	2.31915122106899	0.028158145
Hand Grip Strength	-1.71031418696307	0.056172163
Pairs Matching Test Score	1.13402076987003	0.057561141
BMI	0.819367381913949	0.060829566
Income Category	-0.814611096828761	0.07486036
Number of Hours Slept Per Night	1.57859623037423	0.077959147
Number of Depression Episodes	4.8565312910675	0.091357239
Basal Metabolic Rate	0.701564061956028	0.108464159
Age when left higher education	-0.717055639700312	0.125483647
Bodyfat Percentage	0.617617553997329	0.157636346
Number of Government Health Advice Ignored	0.936985425598994	0.158323287
Waist Circumference	0.600482230168807	0.169461931
Reaction Time when Paying SNAP	1.99284560746826	0.181329914
Mental Health Score	-0.624692928858386	0.254677738
Height	1.59394428485759	0.284997425
WHR corrected for BMI	0.581842308240391	0.287882169
Forced Vital Capacity	-0.530367531543982	0.291322609
Systolic Blood Pressure	0.451437099887695	0.298756283
Forced Expiratory Volume	1.51844030911441	0.308460006
Birthweight	1.48822730493224	0.320048761
Diastolic Blood Pressure	-0.539023380526832	0.3248363
Fluid Intelligence Test Score	-1.41907796512982	0.341398854
Age At Menarche	-0.597002347783648	0.400048963
Hearing Right	0.983083684034577	0.509987615

Supplementary Table 14: Top UK Biobank traits association – *FIBIN BBOX1* Duplication

Pheno	Beta (Inverse Norm)	P
BMI	1.2874568398926	9.09543E-09
Bodyfat Percentage	1.19669540028498	1.80723E-07
Waist Circumference	1.12936386539182	4.62515E-07
Basal Metabolic Rate	1.11872506188444	1.07066E-06
Birthweight	1.0889969382322	0.000180804
Bone Mineral Density	0.820732787819288	0.001272584
General Happiness Score	0.717575954702981	0.056187526
Height	0.433500182384755	0.063579354
Age At Menarche	-0.723242051679829	0.068197977
Income Category	-0.425059622481287	0.072286296
Reaction Time when Paying SNAP	-0.323507526314869	0.103007979
Fluid Intelligence Test Score	0.582796370984299	0.117897265
Mental Health Score	0.334578548562066	0.141846673
Forced Expiratory Volume	-0.311899582017326	0.160578482
Hearing Left	0.501045606262624	0.182694054
Number of Depression Episodes	-0.735141042291542	0.183581979
Hearing Right	0.520201677930546	0.215134471
Forced Vital Capacity	-0.385197413130512	0.218966361
Systolic Blood Pressure	-0.280176574115318	0.247185936
Hand Grip Strength	0.258859793094818	0.284771709
Number of Hours Slept Per Night	0.19356321004384	0.39456631
Age when left higher education	-0.205421416112299	0.403756987
Number of Government Health Advice Ignored	0.174898540037099	0.461449941
Diastolic Blood Pressure	0.152498877143301	0.496054025
Pairs Matching Test Score	-0.135445929659218	0.521072579
WHR corrected for BMI	-0.14621047724507	0.532733176
Townsend Deprivation Index	-0.113209825207801	0.567671383

Supplementary Table 15: Top UK Biobank traits association – *MC4R* region Deletion

Pheno	Beta (Inverse Norm)	P
Age At Menarche	-1.52408194989415	0.009194486
Mental Health Score	-1.01136436856113	0.066043956
Number of Government Health Advice Ignored	-1.17967569648827	0.081571041
Townsend Deprivation Index	0.720526268963964	0.082349696
Hearing Left	-1.06951503626029	0.083094371
Age when left higher education	-1.13956132894036	0.086947419
General Happiness Score	-1.12565046553978	0.115010119
Height	-1.01379497440273	0.131819416
Hand Grip Strength	-0.622717399977821	0.134586732
Forced Vital Capacity	-0.713409631408813	0.140821806
Bone Mineral Density	0.644011148828381	0.182066272
Systolic Blood Pressure	0.634213131009606	0.18367113
Pairs Matching Test Score	-0.721494928015646	0.185473324
WHR corrected for BMI	-0.877334317041033	0.194516555
Number of Hours Slept Per Night	-0.524177543915688	0.207846184
Forced Expiratory Volume	0.695607882422717	0.214354592
Fluid Intelligence Test Score	-0.866635721012498	0.215707417
BMI	0.508298099228124	0.220392978
Waist Circumference	0.668894608084148	0.269674797
Bodyfat Percentage	0.639446287922858	0.291381921
Diastolic Blood Pressure	-0.690656787408137	0.300094057
Basal Metabolic Rate	-0.631314922998035	0.350561941
Reaction Time when Paying SNAP	0.498371636280979	0.410850025
Income Category	0.750733066081982	0.431294166
Birthweight	0.401501790074529	0.468374974
Hearing Right	0.483092369068406	0.499133365
Number of Depression Episodes	-1.79377681681671	0.682913626

Supplementary Table 16: Top UK Biobank traits association – *MC4R* region Duplication

Pheno	Beta (Inverse Norm)	P
Hand Grip Strength	-2.8545618662096	0.001395612
Waist Circumference	-2.90268450847608	0.002635523
Basal Metabolic Rate	-2.72820033090958	0.005194172
Pairs Matching Test Score	2.30936389124103	0.010058217
Mental Health Score	-23.7440918114016	0.014350028
BMI	-2.29787816482944	0.019911956
Hearing Left	-8.24091356965084	0.022470974
Age At Menarche	-4.67100076509518	0.037480058
Fluid Intelligence Test Score	-24.9761568282883	0.038346408
WHR corrected for BMI	-1.78775586552194	0.038818777
Forced Vital Capacity	-12.0506811878732	0.051771394
General Happiness Score	-22.4366865701911	0.062709251
Bodyfat Percentage	-1.72958442502477	0.064630398
Reaction Time when Paying SNAP	1.80926815373641	0.070102942
Height	-1.68778752534211	0.071344816
Hearing Right	-20.9194200406331	0.08281571
Bone Mineral Density	-1.46124015965162	0.108724245
Forced Expiratory Volume	-9.1225041093684	0.140910225
Number of Hours Slept Per Night	1.34254866919566	0.174082628
Number of Government Health Advice Ignored	7.01274828568291	0.217177372
Number of Depression Episodes	7.02702202169166	0.231069631
Income Category	-2.94538127227924	0.264974597
Diastolic Blood Pressure	1.03200285751648	0.265084501
Systolic Blood Pressure	1.00884051698689	0.275956966
Birthweight	0.784791202736823	0.433020341
Townsend Deprivation Index	0.524875148815789	0.575618025
Age when left higher education	-3.07790340913797	0.639012578

Supplementary Table 17: Top UK Biobank traits association – *7q11.23* Deletion

Pheno	Beta (Inverse Norm)	P
Age At Menarche	2.20965127532717	0.001146906
Income Category	-1.50125456532932	0.00186953
Waist Circumference	1.21000431247876	0.005291168
Bone Mineral Density	1.20024687860349	0.00750692
Pairs Matching Test Score	1.0928911716104	0.014254486
Mental Health Score	-1.7853000842924	0.015348088
Bodyfat Percentage	1.31988778399257	0.016876324
Number of Hours Slept Per Night	2.35182848663618	0.018324348
Basal Metabolic Rate	0.987636664948835	0.022845288
Townsend Deprivation Index	0.954896300419198	0.029755281
BMI	0.924527783774362	0.033109819
Fluid Intelligence Test Score	-1.97286735976472	0.042864193
Hand Grip Strength	-0.811254657712104	0.050289493
Birthweight	16.0005667897666	0.051923223
WHR corrected for BMI	1.79120205329927	0.072391569
Forced Vital Capacity	-0.920892990328037	0.084474224
Forced Expiratory Volume	-0.831377462056381	0.104897533
Reaction Time when Paying SNAP	0.874159855528507	0.131746108
Height	1.4556315147367	0.147342702
Age when left higher education	-1.33366005384998	0.185399958
General Happiness Score	-1.15041668902804	0.23757211
Hearing Left	1.14716244267823	0.245769314
Systolic Blood Pressure	-1.16205345677092	0.247364504
Number of Government Health Advice Ignored	0.68264479885369	0.304033655
Number of Depression Episodes	3.19765812268875	0.383616708
Diastolic Blood Pressure	-0.853834204402192	0.395358421
Hearing Right	0.686427133587193	0.453989729

Supplementary Table 18: Top UK Biobank traits association – *7q11.23* Duplication

Pheno	Beta (Inverse Norm)	P
Height	-2.13405418437534	1.90485E-06
Hand Grip Strength	-1.35166355822425	0.001070424
Diastolic Blood Pressure	1.21468233563065	0.001670664
Reaction Time when Paying SNAP	1.34872737506186	0.002657316
Income Category	-1.15604158149114	0.002749175
Systolic Blood Pressure	1.12716788778251	0.003535866
Forced Vital Capacity	-1.43006145018152	0.005007003
Basal Metabolic Rate	-1.2516395152016	0.005213666
Fluid Intelligence Test Score	-16.9406418460931	0.005243192
Townsend Deprivation Index	1.03058485645565	0.00765606
BMI	1.87702927175826	0.008247308
Pairs Matching Test Score	2.4463918984338	0.014142737
Forced Expiratory Volume	-0.922740797461663	0.014863071
Bone Mineral Density	-0.840159320251297	0.042028947
Waist Circumference	1.39952278981851	0.048868914
Number of Government Health Advice Ignored	1.37619112343831	0.051368882
Age when left higher education	-1.39644398158938	0.056004153
Age At Menarche	8.97838295561091	0.082394659
Number of Hours Slept Per Night	0.666862746119331	0.084533111
General Happiness Score	-9.97378898190988	0.100152169
Mental Health Score	0.829112550042091	0.1035549
Hearing Left	-9.3107662558719	0.125096902
Number of Depression Episodes	-1.41074439667684	0.167706596
Bodyfat Percentage	0.962503474163049	0.175547548
WHR corrected for BMI	0.453565011379178	0.239875659
Birthweight	-0.647577000615058	0.362791636
Hearing Right	0.914229998839443	0.364650301

Supplementary Table 19: Top UK Biobank traits association – *3q29* section 1 Deletion

Pheno	Beta (Inverse Norm)	P
Basal Metabolic Rate	1.55374163087194	0.000124379
Bodyfat Percentage	1.56085173791293	0.00024266
BMI	1.54686115909051	0.000386123
Waist Circumference	0.676959996957612	0.001070605
Diastolic Blood Pressure	1.98568445601969	0.001333407
Systolic Blood Pressure	1.93735804839957	0.00174417
Hand Grip Strength	-1.70982150012345	0.011127322
Hearing Right	-10.8457823086025	0.01262258
Forced Expiratory Volume	-0.519788041016521	0.018199421
Mental Health Score	1.50116456033249	0.025895226
WHR corrected for BMI	1.29603936024737	0.036239951
Townsend Deprivation Index	0.601000046460346	0.036635776
Forced Vital Capacity	-0.456779793836993	0.037961296
Number of Depression Episodes	1.1788388067718	0.040680706
Birthweight	1.12684448049525	0.050198825
Hearing Left	1.6464372082718	0.072225582
Bone Mineral Density	0.753459413285597	0.072686491
Number of Government Health Advice Ignored	-1.25563668862448	0.073460416
Pairs Matching Test Score	-1.23291508793158	0.075622202
Age when left higher education	-0.624514896895656	0.096543046
Income Category	-0.715142630318031	0.10592283
Number of Hours Slept Per Night	0.995201459157869	0.139498291
Height	0.397530627221239	0.166882727
Age At Menarche	2.87098713003968	0.206101039
Reaction Time when Paying SNAP	-0.448962616514866	0.234301481
General Happiness Score	1.06589886357795	0.243267086
Fluid Intelligence Test Score	3.63316950620201	0.403219797

Supplementary Table 20: Top UK Biobank traits association – *3q29* section 1 Duplication

Pheno	Beta (Inverse Norm)	P
Height	-2.13405418437534	1.90485E-06
Hand Grip Strength	-1.35166355822425	0.001070424
Diastolic Blood Pressure	1.21468233563065	0.001670664
Reaction Time when Paying SNAP	1.34872737506186	0.002657316
Income Category	-1.15604158149114	0.002749175
Systolic Blood Pressure	1.12716788778251	0.003535866
Forced Vital Capacity	-1.43006145018152	0.005007003
Basal Metabolic Rate	-1.2516395152016	0.005213666
Fluid Intelligence Test Score	-16.9406418460931	0.005243192
Townsend Deprivation Index	1.03058485645565	0.00765606
BMI	1.87702927175826	0.008247308
Pairs Matching Test Score	2.4463918984338	0.014142737
Forced Expiratory Volume	-0.922740797461663	0.014863071
Bone Mineral Density	-0.840159320251297	0.042028947
Waist Circumference	1.39952278981851	0.048868914
Number of Government Health Advice Ignored	1.37619112343831	0.051368882
Age when left higher education	-1.39644398158938	0.056004153
Age At Menarche	8.97838295561091	0.082394659
Number of Hours Slept Per Night	0.666862746119331	0.084533111
General Happiness Score	-9.97378898190988	0.100152169
Mental Health Score	0.829112550042091	0.1035549
Hearing Left	-9.3107662558719	0.125096902
Number of Depression Episodes	-1.41074439667684	0.167706596
Bodyfat Percentage	0.962503474163049	0.175547548
WHR corrected for BMI	0.453565011379178	0.239875659
Birthweight	-0.647577000615058	0.362791636
Hearing Right	0.914229998839443	0.364650301

Supplementary Table 21: Top UK Biobank traits association – *3q29* section 2 Deletion

Pheno	Beta (Inverse Norm)	P
Diastolic Blood Pressure	2.00936179939125	0.000290132
Basal Metabolic Rate	1.10301630979139	0.002389257
Waist Circumference	1.54316710351228	0.00542729
Systolic Blood Pressure	8.15924003735672	0.011690594
Bone Mineral Density	-1.27853929331454	0.014338158
Number of Government Health Advice Ignored	-1.09264447363499	0.016472923
Hand Grip Strength	-1.48777181144305	0.018203532
BMI	1.27444223823957	0.021660825
Number of Hours Slept Per Night	1.1611376044648	0.026151361
Height	0.78610418934282	0.029648874
Income Category	1.68078859745574	0.030251929
Reaction Time when Paying SNAP	1.31388106070481	0.032353607
Forced Expiratory Volume	-1.31799180947328	0.033152824
Mental Health Score	0.749675388606364	0.0367163
Bodyfat Percentage	-1.32802667675714	0.037962075
Pairs Matching Test Score	1.14607415659259	0.038465766
Birthweight	-2.7555498713919	0.047030885
Forced Vital Capacity	-1.19176807900792	0.054077371
Townsend Deprivation Index	0.995203100582651	0.056623952
Age when left higher education	1.08649611123387	0.078928239
General Happiness Score	1.84338405251725	0.079337401
Age At Menarche	5.55447684553796	0.13932996
Number of Depression Episodes	-1.12725598766506	0.15442902
WHR corrected for BMI	-0.767022947485931	0.165976711
Hearing Right	-1.23765068111656	0.239098205
Hearing Left	0.893383985809885	0.244112848
Fluid Intelligence Test Score	0.775849736785917	0.2578305

Supplementary Table 22: Top UK Biobank traits association – *3q29* section 2 Duplication

Chr	Start (Mb) [hg18]	End (Mb) [hg18]	Frequency (%)		BMI			Weight			Height		
			Del	Dup	β	P value	r^2	β	P value	r^2	β	P value	r^2
2	111.3	111.5	0.01	0.02	-	-	-	-8.97	1.33E-05	1.00	-	-	-
2	112.4	112.5	0.03	0.03	-	-	-	-8.77	1.74E-05	1.00	-	-	-
15	20.33	20.6	0.38	0.48	-	-	-	-	-	-	0.74	8.10E-06	0.95
22	17.41	18.69	0.02	0.06	2.11	1.70E-05	0.96	-	-	-	-	-	-
22	19.07	19.33	0.01	0.08	1.85	3.82E-06	1.00	-	-	-	-	-	-

Supplementary Table 23: CNVs associated with one or several of the traits of interest and with a p-value close to the significance level. The first one corresponds to two CNVs in the 2q13 region (111.3-111.5Mb – 112.4-112.5Mb), and whose copy number is negatively associated with weight (section 1: Beta=-8.97kg, P=1.3x10^{-5} – section 2: Beta=-8.77kg, P=1.7x10^{-5}). Larger (1.5-2 Mb) CNVs encompassing this region have been reported in the literature[6] for risk increase of developmental delay[7,8]. The second one is located between the 15q11.2 BP1 and BP2[9,10] (20.33-20.60Mb) and is associated with height (Beta=0.74cm – P=8.1x10^{-6}). It corresponds exactly to the region responsible for the Burnside-Butler syndrome[9], in the proximal interval of Prader-Willi/Angelman. Moreover, it was also found associated with schizophrenia[11]. Finally, two CNVs located in the 22q11.2 region (17.41-18.69Mb and 19.07-19.33Mb) were found to be associated to BMI in our meta-analysis (Beta=2.11kg/m^2 (P=1.7x10^{-5}) and Beta=1.85kg/m^2 (P=3.82x10^{-6}), respectively). They overlap with the location of Di George/22q11.21 duplication syndrome (17.40-19.75Mb)[12,13] with a wide phenotype spectrum. Recently, the deletion, was associated with schizophrenia[11].

Pheno	SNPs	CHR	GENE	min_P_Neff	beta_min	Rsq_min
BMI	rs3888190	16	ATP2A1	0.000189136	-3.07196299	0.756916339
BMI	rs4787491	16	INO80E	5.38E-09	-3.662497866	0.995571108
BMI	rs6567160	18	MC4R	0.00064438	-5.061863809	0.92382604
HT	rs6658763	1	FMO5	4.49E-06	3.459918459	0.933499023
HT	rs11642612	16	FLJ25404	3.39E-10	5.211022089	0.991528719

Supplementary Table 24: CNV association meta-analysis results for known CNVs

Pheno	chr	start	end	P value	Beta	F del (%)	F dup (%)	R square
BMI	16	28700000	28900000	2.32E-05	-3.07196299	0.024	0.041	0.756916339
BMI	16	29514353	30107356	2.39E-09	-3.662497866	0.023	0.026	0.995571108
WT	16	28700000	28900000	2.19E-06	-10.3514665	0.019	0.029	0.935360019

Supplementary Table 25: CNV association meta-analysis results for known SNPs

chr	start	end	Beta BMI			Beta WT			Beta HT			Beta WHR			OR SCZ	
			mirror	del	dup	mirror	del	dup	mirror	del	dup	mirror	del	dup	del	dup
1	145	145.9				6.66	-9.66	6.27	3.46	-6.57					3.80	
3	197.7	197.9				22.55		24.14							INF	
3	198.2	198.4							13.3							
7	72.66	72.75										0.111		0.104		16.10
16	28.73	28.95	-3.07	5.16		-10.35	14.57	-8.01							20.60	
16	29.5	30.11	-3.66	6.15					0.82	-1.31	0.54	-0.041	0.092			9.40
Close to significance CNVs																
15	20.33	20.62							0.74	-1.49					1.80	
22	17.41	18.69	2.11		2.05	5.01		5.09							67.70	0.15
22	19.07	19.33	1.85		1.89	4.98		5.28								

Supplementary Table 26: Significant CNV effects on the traits of interest with on the last column the OR for schizophrenia from the *Marshall et al 2016* paper on association between CNVs and schizophrenia. (human genome build 36)

Cohort	Country	Samples size BMI	Weight	Height	Waist-Hip ratio	Reference
3D	Denmark	942	959	950		https://doi.org/10.1177/0333102415570492
B58C	UK	2334	2334	2334	2334	http://www.b58cgene.sgul.ac.uk/index.php
COROGENE CASE	Finland	1410	1417	1411		http://ije.oxfordjournals.org/content/early/2011/06/02/ije.dyr090.extract
COROGENE CTRL	Finland	1570	1578	1570	1577	http://ije.oxfordjournals.org/content/early/2011/06/02/ije.dyr090.extract
DNBC	Denmark	1590	1588	1612		www.dnbc.dk/
EGCUT	Estonia	7087	7087	7089	6153	http://www.biobank.ee
FAMHS	US	755	755	755	755	https://dsgweb.wustl.edu/PROJECTS/MP1.html
FAMHS1M	US	465	465	465	465	https://dsgweb.wustl.edu/PROJECTS/MP1.html
FINRISK	Finland	13056	13068	13060	13067	http://www.ktl.fi/portal/4168
Generation Scotland	UK	6390	6391	6412	6329	http://www.ed.ac.uk/generation-scotland/
H2000	Finland	1399	1400	1399	1399	http://www.terveys2000.fi/indexe.html
HBCS	Finland	1448	1449	1448	1447	http://www.ktl.fi/portal/english/research_people_programs/health_promotion_and_chronic_disease_prevention/units/diabetes_unit/idefix_study/
Hypergenes	Italy	2930				http://www.hypergenes.eu/
InCHIANTI	Italy	702	706	700	715	http://www.inchiantistudy.net/
LifeLine	Netherlands	11486		11486		http://www.lifelines.nl
LLFS	US	476	476	478		https://dsgweb.wustl.edu/llfs/
LOLIPOP	UK	1243	1243	1243	1243	http://www.lolipopstudy.org/
Mt Sinai BioMe	US	1301	1233			http://icahn.mssm.edu/research/ipm/programs/biome-biobank
PREDICTCVD	Finland	1744	1757	1744	1757	http://www.ktl.fi/portal/4168
QIMR	Australia	7385	7385	7419		http://www.qimrberghofer.edu.au/
SCCS	Switzerland	750				http://www.gastroenterologie.usz.ch/forschung/hepatologie/seiten/unterseite-3.aspx
SSC	US	701				https://sfari.org/resources/autism-cohorts/simons-simplex-collection
TRAILSPOP	Netherlands	1171	1171	1173	1167	http://www.trails.nl/
TwinsUK	UK	1195	1195	1195	1195	http://www.twinsuk.co.uk/
UKBB	UK	119873	119767	116259	119867	https://www.ukbiobank.ac.uk/
YFS	Finland	1758	1759	1763	1774	http://vanha.med.utu.fi/cardio/youngfinnsstudy/
**TOTAL**		**191161**	**175183**	**181965**	**161244**	

Supplementary Table 27: List of all the cohorts participating to this meta-analysis.

Metrics	Definition
Confidence	Likelihood of the most likely CN minus the 2nd most likely one
Length	CNV length in bp
N probes	Number of probes in the CNV
LRR mean	Mean LRR for a given sample
LRR sd	Standard deviation of the LRR
BAF mean	Mean BAF for a given sample
BAF sd	Standard deviation of the BAF
BAF drift	Summarize the proportion of BAF values falling out of the expected BAF (0, 0.5 and 1)
WF	Value measuring the strength of waviness in a given sample
N CNVs	Number of CNVs per sample

Supplementary Table 28: List of the quality metrics extracted from PennCNV and used to calculate the CNV quality score.

Supplementary Notes

Supplementary Note 1. *Example configuration file to run the CNV-calling pipeline*

```
pennCNVpath:    /data/sgg/aurelien/software/pennCNV/penncnv_2
HMMpath:    /data/sgg/aurelien/software/pennCNV/penncnv_2/lib/hhall.hmm
HMMcreate:  0
PFB:  example/pfb/
CompilePFB: 1
GCmod:      my/GC_model/path
UseGCmod:   0
InputData:  1
DATA: example/raw
OUTPUT:     example/results
FormatedPath:     example/formated
Chromosome: 1-22
CNVcall:    1
Cleancall:  1
format:     1
CreateRfile:      1
AssoData:   1
NbCores:    16
PhenoPath:  example/phenotype/phenotype.txt
Phenotype:  example
```

Supplementary Note 2: *Full funding information*

This work was supported by the Leenards Foundation [to Z.K.], the Swiss National Science Foundation [31003A_169929 to Z.K., Sinergia grant CRSII33-133044 to AR], Simons Foundation [SFARI274424 to AR] and SystemsX.ch [51RTP0_151019 to Z.K.]. The EGCUT researchers were funded by EU H2020 grant #692145, #692065, Estonian Research Council Grant IUT20-60, IUT24-6, and European Union through the European Regional Development Fund Project No. 2014-2020.4.01.15-0012 GENTRANSMED. KM is a Jacobs Foundation Research Fellow. A.R.W., H.Y. and T.M.F. are supported by the European Research Council grant: 323195:SZ-245. M.A.T., M.N.W. and A.M. are supported by the Wellcome Trust Institutional Strategic Support Award(WT097835MF). R.B. is funded by the Wellcome Trust and Royal Society grant:104150/Z/14/Z. S.E.J. is funded by the Medical Research Council (grant:MR/M005070/1). R.M.F. is a Sir Henry Dale Fellow (Wellcome Trust and Royal Society grant: 104150/Z/14/Z). J.T is funded by a Diabetes Research and Wellness Foundation Fellowship. Finrisk study was supported by the Academy of Finland (grant numbers 139635,

129494, 129322, 250207, 136895 and 263836), the Finnish Foundation for Cardiovascular Research, and the Sigrid Jusélius Foundation. I.M.H was supported by the BMBF-01ER1206, BMBF-01ER1507. COROGENE was supported by grants from Helsinki University Central Hospital special government funds (EVO #TYH7215, #TKK2012005, #TYH2012209, #TYH2014312), and Finnish Foundation for Cardiovascular research. The EU FP7 under grant agreements nr. 313010 (BBMRI-LPC), nr. 305280 (MIMOmics), and HZ2020 633589 (Ageing with Elegans), The Finnish Academy grant no. 269517, The Yrjö Jahnsson Foundation. M.D.T was supported by the MRC fellowships: G0501942 and G0902313. This article presents independent research funded partially by the National Institute for Health Research (NIHR) The views expressed are those of the author(s) and not necessarily those of the NHS, the NIHR or the UK Department of Health. We acknowledge use of phenotype and genotype data from the British 1958 Birth Cohort DNA collection, funded by the Medical Research Council grant G0000934 and the Wellcome Trust grant 068545/Z/02. Genotyping for the B58C-WTCCC subset was funded by the Wellcome Trust grant 076113/B/04/Z. The B58C-T1DGC genotyping utilized resources provided by the Type 1 Diabetes Genetics Consortium, a collaborative clinical study sponsored by the National Institute of Diabetes and Digestive and Kidney Diseases (NIDDK), National Institute of Allergy and Infectious Diseases (NIAID), National Human Genome Research Institute (NHGRI), National Institute of Child Health and Human Development (NICHD), and Juvenile Diabetes Research Foundation International (JDRF) and supported by U01 DK062418. B58C-T1DGC GWAS data were deposited by the Diabetes and Inflammation Laboratory, Cambridge Institute for Medical Research (CIMR), University of Cambridge, which is funded by Juvenile Diabetes Research Foundation International, the Wellcome Trust and the National Institute for Health Research Cambridge Biomedical Research Centre; the CIMR is in receipt of a Wellcome Trust Strategic Award (079895). The B58C-GABRIEL genotyping was supported by a contract from the European Commission Framework Programme 6 (018996) and grants from the French Ministry of Research. The Young Finns Study has been financially supported by the Academy of Finland: grants 286284, 134309 (Eye), 126925, 121584, 124282, 129378 (Salve), 117787 (Gendi), and 41071 (Skidi); the Social Insurance Institution of Finland; Kuopio, Tampere and Turku University Hospital Medical Funds (grant X51001); Juho Vainio Foundation;Paavo Nurmi Foundation;Finnish Foundation of Cardiovascular Research ; Finnish Cultural Foundation; Tampere Tuberculosis Foundation;Emil Aaltonen Foundation;Yrjö Jahnsson Foundation;and

Signe and Ane Gyllenberg Foundation. The expert technical assistance in the statistical analyses by Irina Lisinen is gratefully acknowledged. XL is supported by the Nordic Center of Excellence in Health-Related e-Sciences. The Danish National Biobank is supported by the Novo Nordisk Foundation. The study was partly based on samples from the Danish National Birth Cohort, which was established with the support of a major grant from the Danish National Research Foundation with additional support from the Danish Pharmacists' Fund, the Egmont Foundation, the March of Dimes Birth Defects Foundation, the Augustinus Foundation and the Health Fund of the Danish Health Insurance Societies. BF was supported by an Oak Foundation fellowship. This study was supported by the National Health and Medical Research Council of Australia [grant numbers 403981, 1021105, 003209, 82893, and 572613] and the Canadian Institutes of Health Research [grant number MOP-82893]. The authors gratefully acknowledge the following institutes for providing funding for Core Management of the Raine Study: The University of Western Australia (UWA), Curtin University, the Raine Medical Research Foundation, the UWA Faculty of Medicine, Dentistry and Health Sciences, the Telethon Kids Institute, the Women and Infants Research Foundation and Edith Cowan University. This work was supported by resources provided by the Pawsey Supercomputing Centre with funding from the Australian Government and Government of Western Australia. The LOLIPOP study is supported by the National Institute for Health Research (NIHR) Comprehensive Biomedical Research Centre Imperial College Healthcare NHS Trust, the British Heart Foundation (SP/04/002), the Medical Research Council (G0601966,G0700931), the Wellcome Trust (084723/Z/08/Z), the NIHR (RP-PG-0407-10371), European Union FP7 (EpiMigrant, 279143) and Action on Hearing Loss (G51). TRAILS has been financially supported by various grants from the Netherlands Organization for Scientific Research NWO (Medical Research Council program grant GB-MW 940-38-011; ZonMW Brainpower grant 100-001-004; ZonMw Risk Behavior and Dependence grants 60-60600-97-118; ZonMw Culture and Health grant 261-98-710; Social Sciences Council medium-sized investment grants GB-MaGW 480-01-006 and GB-MaGW 480-07-001; Social Sciences Council project grants GB-MaGW 452-04-314 and GB-MaGW 452-06-004; NWO large-sized investment grant 175.010.2003.005; NWO Longitudinal Survey and Panel Funding 481-08-013), the Dutch Ministry of Justice (WODC), the European Science Foundation (EuroSTRESS project FP-006), Biobanking and Biomolecular Resources Research Infrastructure BBMRI-NL (CP 32), and the participating universities. Statistical analyses were

carried out on the Genetic Cluster Computer (http://www.geneticcluster.org) hosted by SURFsara and financially supported by the Netherlands Scientific Organization (NWO 480-05-003 PI: Posthuma) along with a supplement from the Dutch Brain Foundation and the VU University Amsterdam. 3D has been supporte by the LuF Grant number: R155-2014-1724. InCHIANTI has been supported by the Wellcome Trust Institutional Strategic Support Award(WT097835MF). QIMR was supported by the Australian National Health and Medical Research Council (NHMRC grants 241944, 339446, 389875, 389891, 389892, 389938, 442915, 442981, 496739, 496688, 552485, 613672, 613674, 619667, 1049472, 1050208, 1075175, 1078399), the U.S. National Institute of Health (grants AA07535, AA10248, AA014041, AA13320, AA13321, AA13326 and DA12854) and the Australian Research Council (ARC grant DP0770096). FamHS was supported by NIDDK-R01DK089256, NHLBI-R01HL117078. LLFS was supported by NIA-U01AG023746. The KORA study was initiated and financed by the Helmholtz Zentrum München – German Research Center for Environmental Health, which is funded by the German Federal Ministry of Education and Research (BMBF) and by the State of Bavaria. Furthermore, KORA research was supported within the Munich Center of Health Sciences (MC-Health), Ludwig-Maximilians-Universität, as part of LMUinnovativ.

Supplementary References

1. Locke, A.E. *et al.* Genetic studies of body mass index yield new insights for obesity biology. *Nature* **518**, 197-206 (2015).
2. Wood, A.R. *et al.* Defining the role of common variation in the genomic and biological architecture of adult human height. *Nat Genet* **46**, 1173-86 (2014).
3. Peterson, R.E. *et al.* On the association of common and rare genetic variation influencing body mass index: a combined SNP and CNV analysis. *BMC Genomics* **15**, 368 (2014).
4. Kaur, Y., de Souza, R.J., Gibson, W.T. & Meyre, D. A systematic review of genetic syndromes with obesity. *Obes Rev* (2017).
5. McKusick-Nathans Institute of Genetic Medicine, J.H.U.B., MD). Online Mendelian Inheritance in Man, OMIM®. (2016).
6. Rudd, M.K. *et al.* Segmental duplications mediate novel, clinically relevant chromosome rearrangements. *Hum Mol Genet* **18**, 2957-62 (2009).
7. Coe, B.P. *et al.* Refining analyses of copy number variation identifies specific genes associated with developmental delay. *Nat Genet* **46**, 1063-71 (2014).
8. Hladilkova, E. *et al.* A recurrent deletion on chromosome 2q13 is associated with developmental delay and mild facial dysmorphisms. *Mol Cytogenet* **8**, 57 (2015).
9. Burnside, R.D. *et al.* Microdeletion/microduplication of proximal 15q11.2 between BP1 and BP2: a susceptibility region for neurological dysfunction including developmental and language delay. *Hum Genet* **130**, 517-28 (2011).
10. Cox, D.M. & Butler, M.G. The 15q11.2 BP1-BP2 microdeletion syndrome: a review. *Int J Mol Sci* **16**, 4068-82 (2015).
11. Contribution of copy number variants to schizophrenia from a genome-wide study of 41,321 subjects. *Nat Genet* (2016).
12. McDonald-McGinn, D.M., Emanuel, B.S. & Zackai, E.H. 22q11.2 Deletion Syndrome. in *GeneReviews(R)* (eds. Pagon, R.A. *et al.*) (Seattle (WA), 1993).
13. Robin, N.H. & Shprintzen, R.J. Defining the clinical spectrum of deletion 22q11.2. *J Pediatr* **147**, 90-6 (2005).
